# Positive Autism Screening Rates in Toddlers Born During the COVID-19 Pandemic

**DOI:** 10.1001/jamanetworkopen.2024.35005

**Published:** 2024-09-23

**Authors:** Morgan R. Firestein, Angela Manessis, Jennifer M. Warmingham, Ruiyang Xu, Yunzhe Hu, Morgan A. Finkel, Margaret Kyle, Maha Hussain, Imaal Ahmed, Andréane Lavallée, Ana Solis, Vitoria Chaves, Cynthia Rodriguez, Sylvie Goldman, Rebecca A. Muhle, Seonjoo Lee, Judy Austin, Wendy G. Silver, Kally C. O’Reilly, Jennifer M. Bain, Anna A. Penn, Jeremy Veenstra-VanderWeele, Melissa S. Stockwell, William P. Fifer, Rachel Marsh, Catherine Monk, Lauren C. Shuffrey, Dani Dumitriu

**Affiliations:** 1 Department of Psychiatry, Columbia University Irving Medical Center, New York, New York; 2 Teachers College, Columbia University, New York, New York; 3 Department of Pediatrics, Columbia University Irving Medical Center, New York, New York; 4 NewYork-Presbyterian Morgan Stanley Children’s Hospital, New York; 5 Department of Biostatistics, Mailman School of Public Health, Columbia University Irving Medical Center, New York, New York; 6 New York State Psychiatric Institute, New York; 7 Department of Population and Family Health, Mailman School of Public Health, Columbia University Irving Medical Center, New York, New York; 8 Department of Neurology, Division of Child Neurology, Columbia University Irving Medical Center, New York, New York; 9 Department of Obstetrics and Gynecology, Columbia University Irving Medical Center New York, New York; 10 Department of Child and Adolescent Psychiatry, New York University Grossman School of Medicine, New York

## Abstract

**Importance:**

Stress and viral illness during pregnancy are associated with neurodevelopmental conditions in offspring. Autism screening positivity for children born during the pandemic remains unknown.

**Objective:**

To examine associations between prenatal exposure to the pandemic milieu and maternal SARS-CoV-2 infection with rates of positive Modified Checklist for Autism in Toddlers, Revised (M-CHAT-R) screenings.

**Design, Setting, and Participants:**

Data for this cohort study were drawn from the COVID-19 Mother Baby Outcomes (COMBO) Initiative. M-CHAT-R scores obtained from children aged 16 to 30 months during routine clinical care at Columbia University Irving Medical Center in New York City were abstracted from electronic health records (EHRs) for children born between January 2018 and September 2021 (COMBO-EHR cohort). Separately, the M-CHAT-R was administered at 18 months for children born between February 2020 and September 2021 through a prospective longitudinal study (COMBO-RSCH cohort). Prenatal pandemic exposure (birth after March 1, 2020) and maternal SARS-CoV-2 status during pregnancy was determined through EHRs. Data were analyzed from March 2022 to June 2024.

**Exposures:**

Prenatal exposures to the pandemic milieu and maternal SARS-CoV-2 infection.

**Main Outcomes and Measures:**

The primary outcome was rate of positive M-CHAT-R screenings. For all primary analyses, unadjusted χ^2^ tests and adjusted logistic regression models were performed.

**Results:**

The COMBO-EHR cohort included 1664 children (442 born before the pandemic and 1222 born during the pandemic; 997 SARS-CoV-2 unexposed, 130 SARS-CoV-2 exposed, and 95 with unknown SARS-CoV-2 exposure status), of whom 266 (16.0%) were Black, 991 (59.6%) were Hispanic, 400 (24.0%) were White, 1245 (74.8%) were insured through Medicaid, 880 (52.9%) were male, and 204 (12.3%) were born prematurely. The COMBO-RSCH cohort included 385 children (74 born before the pandemic and 311 born during the pandemic; 201 SARS-CoV-2 unexposed, 101 SARS-CoV-2 exposed, and 9 with unknown SARS-CoV-2 exposure status), of whom 39 (10.1%) were Black, 168 (43.6%) were Hispanic, 157 (40.8%) were White, 161 (41.8%) were insured through Medicaid, 222 (57.7%) were male, and 38 (9.9%) were born prematurely. Prenatal pandemic exposure was not associated with a higher positive M-CHAT-R screening rate in either the COMBO-EHR or COMBO-RSCH cohort. Prenatal exposure to maternal SARS-CoV-2 infection was associated with a lower rate of M-CHAT-R positivity in the COMBO-EHR cohort (12.3% [16 children] vs 24.0% [239 children]; adjusted odds ratio, 0.40; 95% CI, 0.22-0.68; *P* = .001), but no association was found in the COMBO-RSCH cohort (12.9% [13 children] vs 19.9% [40 children]; adjusted odds ratio, 0.51; 95% CI, 0.24-1.04; *P* = .07).

**Conclusions and Relevance:**

In this cohort study of 2 groups of children with prenatal pandemic exposure and/or exposure to maternal SARS-CoV-2 infection, neither exposure was associated with greater M-CHAT-R positivity.

## Key Points

**Question:**

Are prenatal exposures to the COVID-19 pandemic milieu and/or maternal SARS-CoV-2 infection associated with higher positivity rates on the Modified Checklist for Autism in Toddlers, Revised (M-CHAT-R) parent-report autism screener among children aged 16 to 30 months?

**Findings:**

This cohort study of approximately 2000 children in New York City revealed no significant association between prenatal exposure to the pandemic milieu and M-CHAT-R positivity. Prenatal exposure to maternal SARS-CoV-2 infection was associated with lower rates of M-CHAT-R positivity.

**Meaning:**

These findings suggest prenatal exposures to the pandemic milieu and maternal SARS-CoV-2 infection were not associated with greater M-CHAT-R positivity.

## Teaser

This cohort study evaluates associations between prenatal exposure to maternal SARS-CoV-2 infection with rates of positive Modified Checklist for Autism in Toddlers, Revised screenings.

## Introduction

Longitudinal research is needed to determine long-term consequences of early exposure to both the SARS-CoV-2 virus and the COVID-19 pandemic milieu, especially those affecting the youngest generations.^1,2,3^ Most reports provide reassuring results suggesting no association between prenatal exposure to maternal SARS-CoV-2 infection and child neurodevelopment.^4,5,6,7,8^ One research group^9,10^ found that a higher proportion of prenatally exposed male infants received *International Statistical Classification of Diseases and Related Health Problems, Tenth Revision* codes for speech disorders at age 12 months, but not at 18 months. Furthermore, a study^11^ of infants born during previous pandemics has shown that neurodevelopmental decrements frequently emerge in later years, necessitating additional follow-up.

Beyond prenatal SARS-CoV-2 exposure, children born during the pandemic experienced an unusual environment during infancy and early childhood secondary to societal changes and restrictions. Several reports^4,6,7,12^ from diverse populations revealed neurodevelopmental differences between children born during the pandemic compared with those born before the pandemic.

The COVID-19 pandemic, therefore, presents a 2-pronged mechanism through which children born during the pandemic may be at greater risk for neurodevelopmental conditions, such as autism spectrum disorders.^6,13^ Maternal prenatal psychological distress has been implicated in increased neurodevelopmental risk through a fetal programming framework.^14,15^ Separately, maternal immune activation (MIA) is among the putative mechanisms through which prenatal SARS-CoV-2 exposure has been hypothesized to impact offspring neurodevelopment. Previous outbreaks of human coronaviruses (such as SARS and Middle East Respiratory Syndrome) suggest that severe infection during pregnancy may negatively affect both maternal health and child well-being, possibly through MIA.^13,16^

Given the benefits of early intervention,^17,18^ special attention should be directed toward evaluating the association between fetal exposure to maternal SARS-CoV-2 infection, the COVID-19 pandemic milieu, and child neurodevelopment. Children who were in utero during peak periods of the pandemic are reaching the age at which early indicators of autism emerge. We compared scores on the parent-report Modified Checklist for Autism in Toddlers, Revised (M-CHAT-R) screener for children born before and during the pandemic and those with and without prenatal SARS-CoV-2 exposure. Data were collected as part of the COVID-19 Mother Baby Outcomes (COMBO) Initiative from a diverse university-based medical center in New York, New York (New York City [NYC]), and include clinical M-CHAT-R scores recorded in electronic health records (EHRs) and M-CHAT-R scores obtained for research purposes (RSCH).

## Methods

### Study Design and Participants

Data for this cohort study were drawn from the COMBO Initiative for children born at Columbia University Irving Medical Center (CUIMC)–affiliated NewYork-Presbyterian (NYP) Morgan Stanley Children’s Hospital and NYP Allen Pavilion Hospital in NYC. COMBO includes an EHR group (COMBO-EHR cohort) with a waiver of consent and a prospective research group (COMBO-RSCH cohort) with ongoing enrollment and consent of mother-child dyads (eFigure 1 in Supplement 1). For consistency, demographic and clinical variables were acquired from EHRs for both cohorts through a combination of automated abstraction and manual review. As part of routine clinical care, all CUIMC/NYP-affiliated pediatric clinics began universally administering the M-CHAT-R at 18-month and 24-month well child checks in February 2020. Compliance with universal screening is 45% (Evelyn Berger-Jenkins, MD, MPH, personal communication, June 4, 2024). Separately, mothers enrolled in COMBO-RSCH complete the M-CHAT-R at the 18-month study follow-up via secure online REDCap surveys. All study procedures have been reviewed and approved by the CUIMC institutional review board. This study followed the Strengthening the Reporting of Observational Studies in Epidemiology (STROBE) reporting guidelines.

### COMBO-EHR Cohort

Children born between January 2018 and September 2021 who continued care within the CUIMC/NYP primary care network (approximately one-quarter of children born at CUIMC/NYP) and had 1 or more valid M-CHAT-R scores in their EHR were identified (1747 children) through automated extraction. For children with 2 or more valid M-CHAT-R scores, we used the most recent score. There were no group differences in how many scores children had documented in their EHR. We excluded 80 children for M-CHAT-R completion outside valid age range. We excluded an additional 3 children who received a total M-CHAT-R score of 17 resulting from all items being marked as no, suggesting invalid parental report.

### COMBO-RSCH Cohort

At the time of this analysis, COMBO had enrolled 907 mother-child dyads with infants born at CUIMC/NYP starting February 2020, of whom 386 children born between February 2020 and September 2021 had an M-CHAT-R completed for research purposes. One child was excluded for completion outside valid age range.

### Prenatal Pandemic Exposure

We defined the onset of the pandemic in NYC as March 1, 2020. The COMBO-EHR cohort includes 442 children born before the pandemic and 1222 children born during the pandemic. The COMBO-RSCH cohort includes 74 children born before the pandemic and 311 children born during the pandemic. Of note, all children in both cohorts, including the COMBO-EHR group born before the pandemic, were assessed during the pandemic as universal M-CHAT-R screening at pediatric clinics coincided with the pandemic onset for unrelated reasons.

### Determination of Prenatal Maternal SARS-CoV-2 Infection

Maternal SARS-CoV-2 status during pregnancy was determined through EHR abstraction and review for both cohorts. Detailed descriptions of our methods for determining maternal SARS-CoV-2 status during pregnancy have been reported previously^4,5^ and are outlined in eMethods 1 and eFigure 2 in Supplement 1. Briefly, CUIMC/NYP implemented universal SARS-CoV-2 screening of all delivering patients by nasopharyngeal polymerase chain reaction (PCR) on March 22, 2020 and by serological testing on July 20, 2020. For children born during the pandemic, 1189 mothers (97.3%) in the COMBO-EHR and 311 mothers (100%) in the COMBO-RSCH had PCR and/or serological testing. Children born before November 1, 2020, were considered exposed during pregnancy if their mother had a positive PCR and/or serological test during pregnancy or at delivery. If their mother had a negative PCR test and no available serological test result, they were classified as unexposed. For children born after November 1, 2020, a positive serological test during pregnancy could indicate a prepregnancy infection. Therefore, these children were considered exposed during pregnancy only if their mother additionally had a positive PCR and/or antigen test and/or COVID-19 symptoms during pregnancy. Children were classified as unexposed if their mother had no or negative serological testing during pregnancy. Children were excluded from analysis if timing of maternal infection was undetermined (no PCR or antigen testing during pregnancy and positive serological testing without symptoms during pregnancy) or confirmed to have occurred prepregnancy by PCR or serological testing. In the COMBO-EHR cohort, 33 were excluded for lacking PCR and serological testing. Additionally, 62 in the COMBO-EHR cohort and 9 in the COMBO-RSCH cohort were excluded because their SARS-CoV-2 exposure status was undetermined or occurred prepregnancy.

### Modified Checklist for Autism in Toddlers, Revised

The M-CHAT-R was developed to assess risk for autism on the basis of parent report of their child’s development between 16 to 30 months of age and consists of 20 yes-or-no items that focus on neurodevelopmental milestones and early autism symptoms.^19^ Total scores are calculated and categorized into ranges of low-risk (0-2), medium-risk (3-7), and high-risk (8-20). To improve power, medium-risk and high-risk scores were collapsed and defined as screening positive. The M-CHAT-R is a validated screener with high sensitivity and specificity as a measure for likelihood of autism and is widely adopted by clinicians.^20,21^ Screening positive prompts further clinical follow-up and investigation. The M-CHAT-R was administered in English or Spanish depending on mother’s preference.

### Statistical Analysis

Statistical analyses were conducted in R statistical software version 4.3.1 (R Project for Statistical Computing).^22^ The experimental design (forced-choice online surveys and EHR review) resulted in no missingness except for race and ethnicity, for which unknown was included as a category. For all primary analyses, we performed unadjusted χ^2^ tests and adjusted logistic regression models. For subgroup and sensitivity analyses, only adjusted logistic regression models were performed. Adjusted models included covariates identified a priori as potential confounders associated with either or both the outcome (M-CHAT-R score) and SARS-CoV-2 exposure: child’s age at assessment, sex assigned at birth, mode of delivery, gestational age at delivery, maternal age at delivery, insurance type as a proxy for socioeconomic status (SES) (Medicaid vs commercial), and maternal race and ethnicity (determined through abstraction of self-reported data in EHR). Options for ethnicity included Hispanic or Latino or Spanish Origin (hereafter, Hispanic), not Hispanic or Latino or Spanish Origin (hereafter not Hispanic), declined, and unknown. Options for race included American Indian or Alaska Native, Asian, Black or African American, Native Hawaiian or Other Pacific Islander, White, other combinations not described, declined, and unknown. Race was assessed in this study because of its known association with M-CHAT-R positivity rates. To examine potential demographic differences between groups, comparisons were performed using χ^2^ tests for binomial variables and Mann-Whitney tests for continuous variables. For adjusted logistic analyses, we report adjusted odds ratios (aORs) with 95% CIs due to a priori anticipated positivity rate of approximately 10%.^23^ Because prematurity, male sex, Hispanic ethnicity, and low SES are associated with higher positive screenings for neurodevelopmental disorders,^24,25,26^ separate subgroup analyses were performed for preterm, full-term, male, female, Hispanic, not Hispanic, Medicaid-insured, and commercially insured children. Sensitivity analyses were conducted excluding children born in March 2020 who had a short duration of prenatal exposure to the pandemic for prenatal pandemic exposure analyses and excluding cases with increased risk of misclassification of SARS-CoV-2 exposure due to missing serological testing for prenatal SARS-CoV-2 exposure analyses. Significance was set at *P* < .05. Data were analyzed from March 2022 to June 2024.

## Results

### Cohort Characteristics

The COMBO-EHR cohort consisted of 1664 children (442 born before the pandemic and 1222 born during the pandemic). The median (range) maternal age at delivery was 30 (16-56) years. A total of 8 mothers (0.5%) self-identified as American Indian or Alaska Native, 65 (3.9%) as Asian, 266 (16.0%) as Black, 400 (24.0%) as White, 608 (36.5%) as other combinations not described, 180 (10.8%) declined to answer, and 137 (8.2%) were of unknown race. A total of 991 mothers (59.6%) self-identified as Hispanic, 398 (23.9%) as non-Hispanic, 95 (5.7%) declined to answer, and 180 (10.8%) were of unknown ethnicity. A total of 204 infants (12.3%) were born prematurely, and 880 children (52.9%) were male. In the group born during the pandemic, 95 dyads had an undetermined or prepregnancy infection and were, therefore, excluded from prenatal SARS-CoV-2 exposure analyses, resulting in 997 unexposed and 130 exposed children. The group born before the pandemic had a higher maternal age (Mann-Whitney U test [*U*] = 296 990; *P* = .002), lower infant gestational age (*U* = 249 410; *P* = .02), and a higher percentage of male children (χ^2^_1_ = 6.97; *P* = .01). Characteristics of the COMBO-EHR cohort and other small but significant group differences are summarized in Table 1.

**Table 1.** COMBO Electronic Health Record Cohort Characteristics^a^

Variable	Overall sample, No. (%) (N = 1664)	Born before vs during pandemic				SARS-CoV-2 unexposed vs exposed			
		Participants, No. (%)		Test statistic	*P* value	Participants, No. (%)		Test statistic	*P* value
		Before pandemic (n = 442)	During pandemic (n = 1222)			Unexposed (n = 997)	Exposed (n = 130)		
Maternal characteristics									
Age at delivery, median (range), y	30 (16-56)	31 (16-56)	30 (17-55)	296 990^b^	.002	30 (17-55)	28 (17-43)	70 947^b^	.08
Insurance									
Commercial	419 (25.18)	167 (37.78)	252 (20.62)	49.83^c^	<.001	213 (21.36)	24 (18.46)	0.42^c^	.52
Medicaid	1245 (74.82)	275 (62.22)	970 (79.38)			784 (78.64)	106 (81.54)		
Race^d^									
American Indian/Alaska Native	8 (0.48)	2 (0.45)	6 (0.49)			3 (0.30)	1 (0.77)		
Asian	65 (3.91)	30 (6.79)	35 (2.86)	597.91^c^	<.001	33 (3.31)	2 (1.54)	8.63^c^	.12
Black or African American	266 (15.99)	71 (16.06)	195 (15.96)			166 (16.65)	18 (13.85)		
Native Hawaiian or other Pacific Islander	0	0	0			0	0		
White	400 (24.04)	156 (35.29)	244 (19.97)			200 (20.06)	21 (16.15)		
Other combinations not described	608 (36.54)	30 (6.79)	578 (47.30)			460 (46.14)	76 (58.46)		
Declined	180 (10.82)	16 (3.62)	164 (13.42)			135 (13.54)	12 (9.23)		
Unknown	137 (8.23)	137 (31.00)	0			0	0		
Ethnicity									
Hispanic or Latino/a/x or Spanish	991 (59.56)	144 (32.58)	847 (69.31)	596.41^c^	<.001	669 (67.10)	105 (80.77)	12.31^c^	.002
Not Hispanic or Latino/a/x or Spanish	398 (23.92)	114 (25.79)	284 (23.24)			247 (24.77)	23 (17.69)		
Declined	95 (5.71)	4 (0.90)	91 (7.45)			81 (8.12)	2 (1.54)		
Unknown	180 (10.82)	180 (40.72)	0			0	0		
Infant characteristics									
Age at M-CHAT-R, median (range), mo	23.15 (16.03-30.00)	24.48 (16.26-30.00)	21.42 (16.03-30.00)	383 466^b^	<.001	21.42 (16.03-30.00)	23.36 (16.13-28.65)	57 762^b^	.04
Gestational age at birth, median (range), wk	39.00 (23.00-41.57)	39.00 (23.00-41.29)	39.14 (23.86-41.57)	249 410^b^	.02	39.14 (24.14-41.57)	39.00 (24.71-41.29)	68 099^b^	.35
Preterm birth (<37 wk)	204 (12.26)	43 (9.73)	161 (13.18)	3.27^c^	.07	125 (12.54)	20 (15.38)	0.60^c^	.44
Sex									
Female	784 (47.12)	184 (41.63)	600 (49.10)	6.97^c^	.01	490 (49.15)	65 (50.00)	0.01^c^	.93
Male	880 (52.88)	258 (58.37)	622 (50.90)			507 (50.85)	65 (50.00)		

^a^ All statistical tests were 2-sided.

^b^ Mann-Whitney U test (*U*) for continuous variables.

^c^ Comparisons performed using χ^2^ test for categorical variables.

^d^ Maternal race categories are drawn verbatim from electronic health records.

The COMBO-RSCH cohort consisted of 385 children (74 born before the pandemic and 311 born during the pandemic). The median (range) maternal age at delivery was 32 (18-46) years. A total of 3 mothers (0.8%) self-identified as American Indian or Alaska Native, 13 (3.4%) as Asian, 39 (10.1%) as Black, 1 (0.3%) as Native Hawaiian or other Pacific Islander, 157 (40.8%) as White, 109 (28.3%) as other combinations not described, 63 (16.4%) declined to answer, and 0 (0%) were of unknown race. A total of 168 (43.6%) mothers self-identified as Hispanic, 163 (42.3%) as not Hispanic, 53 (13.8%) declined to answer, and 1 (0.3%) was of unknown ethnicity. A total of 38 infants (9.9%) were born prematurely and 222 children (57.7%) were male. In the group born during the pandemic, 9 dyads had an undetermined or prepregnancy infection and were, therefore, excluded from SARS-CoV-2 exposure analyses, resulting in 201 unexposed and 101 exposed children. Characteristics of the COMBO-RSCH cohort and small but significant group differences are summarized in Table 2.

**Table 2.** COMBO Research Cohort Characteristics^a^

Variable	Overall sample, No. (%) (n = 385)	Born before vs during pandemic				SARS-CoV-2 unexposed vs exposed			
		Participants, No. (%)		Test statistic	*P* value	Participants, No. (%)		*P* value	Test statistic
		Before pandemic (n = 74)	During pandemic (n = 311)			Unexposed (n = 201)	Exposed (n = 101)		
Maternal characteristics									
Age at delivery, median (range), y	32 (18-46)	32 (21-44)	32 (18-46)	10 672^b^	.33	32 (18-46)	32 (20-44)	10 672^b^	.46
Insurance									
Commercial	224 (58.18)	44 (59.46)	180 (57.88)	0.01^c^	.91	118 (58.71)	58 (57.43)	0.01^c^	.93
Medicaid	161 (41.82)	30 (40.54)	131 (42.12)			83 (41.29)	43 (42.57)		
Race^d^									
American Indian or Alaska Nation	3 (0.78)	0	3 (0.96)	6.32^c^	.39	2 (1.00)	1 (.99)	4.37^c^	.63
Asian	13 (3.38)	2 (2.70)	11 (3.54)			9 (4.48)	2 (1.98)		
Black or African American	39 (10.13)	12 (16.22)	27 (8.68)			17 (8.46)	9 (8.91)		
Native Hawaiian or Other Pacific Islander	1 (0.26)	0	1 (0.32)			1 (0.50)	0		
White	157 (40.78)	33 (44.59)	124 (39.87)			85 (42.29)	36 (35.64)		
Other combinations not described	109 (28.31)	16 (21.62)	93 (29.90)			53 (26.37)	36 (35.64)		
Declined	63 (16.36)	11 (14.86)	52 (16.72)			34 (16.92)	17 (16.83)		
Unknown	0	0	0			0	0		
Ethnicity									
Hispanic or Latino/a/x or Spanish	168 (43.64)	29 (39.19)	139 (44.69)	1.02^c^	.80	79 (39.30)	53 (52.48)	7.13^c^	.07
Not Hispanic or Latino/a/x or Spanish	163 (42.34)	34 (45.95)	129 (41.48)			91 (45.27)	36 (35.64)		
Declined	53 (13.77)	11 (14.86)	42 (13.50)			31 (15.42)	11 (10.89)		
Unknown	1 (0.26)	0	1 (0.32)			0	1 (.99)		
Infant characteristics									
Age at M-CHAT-R, median (range), mo	18.48 (16.53-27.01)	18.60 (17.80-20.80)	18.10 (16.53-27.01)	15 052^b^	<.001	18.20 (17.50-27.01)	18.01 (16.53-23.90)	11 124^b^	.17
Gestational age at birth, median (range), wk	39.14 (30.57-41.57)	39.30 (30.57-41.29)	39.14 (31.60-41.57)	12 387^b^	.31	39.14 (33.10-41.57)	39.00 (31.60-41.37)	10 805^b^	.36
Preterm birth (<37 wk)	38 (9.87)	6 (8.11)	32 (10.29)	0.12^c^	.73	15 (7.46)	12 (11.88)	1.12^c^	.29
Sex									
Female	163 (42.34)	33 (44.59)	130 (41.80)	0.09^c^	.76	82 (40.80)	45 (44.55)	0.25^c^	.62
Male	222 (57.66)	41 (55.41)	181 (58.20)			119 (59.20)	56 (55.45)		

Abbreviation: M-CHAT-R, Modified Checklist for Autism in Toddlers, Revised.

^a^ All statistical tests were 2-sided.

^b^ Mann-Whitney test (U) for continuous variables.

^c^ Comparisons performed using χ^2^ test for categorical variables.

^d^ Maternal race categories were drawn verbatim from electronic health records.

Group differences were accounted for in all fully adjusted models for both cohorts. Additional analyses comparing the COMBO-EHR and COMBO-RSCH cohorts are described in eMethods 2 in Supplement 1.

### Prenatal Pandemic Exposure and M-CHAT-R Positive Screening: COMBO-EHR Cohort

There was no difference in the proportion of M-CHAT-R positive screenings between children born before the pandemic and those born during the pandemic (χ^2^_1_ = 0.03; *P* = .87). Specifically, 100 children (22.6%) born before the pandemic and 283 children (23.2%) born during the pandemic screened positive (Figure, A). The lack of an association was maintained in a fully adjusted model (aOR, 0.75; 95% CI, 0.52-1.08; *P* = .12) (Table 3). A sensitivity analysis excluding children with minimal prenatal pandemic exposure (born in March 2020) was also nonsignificant (eTable 1 in Supplement 1). No associations were observed in adjusted subgroup analyses of preterm, full-term, male, female, Hispanic, not Hispanic, Medicaid-insured, or commercially insured children (eTable 2, eTable 3, eTable 4, and eTable 5 in Supplement 1).

**Figure.**
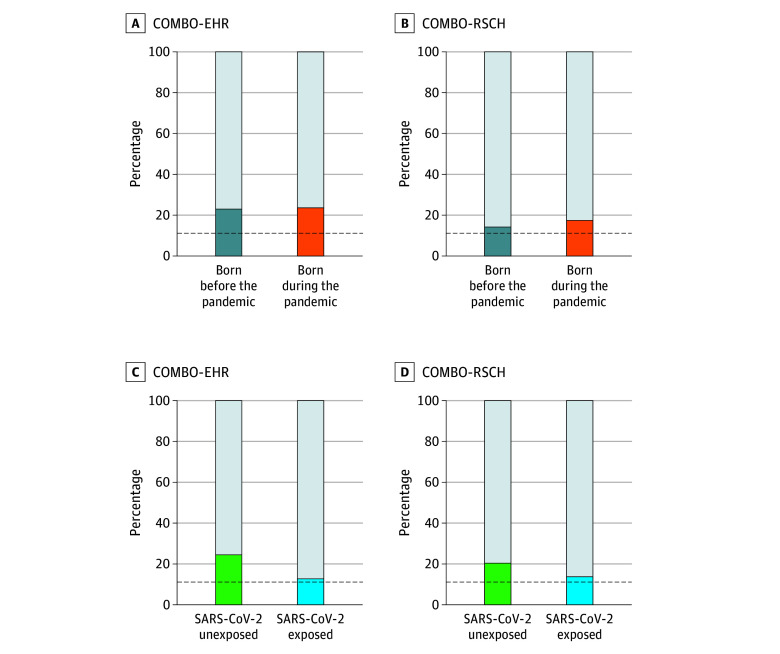
Percentage of Children Screening Positive on the Modified Checklist for Autism in Toddlers, Revised in the Electronic Health Record (COMBO-EHR) and Research (COMBO-RSCH) Datasets Dashed black lines indicate previously reported rates of positivity in low-risk (approximately 10%), US samples.

**Table 3.** Comparison of Rates of Positive Modified Checklist for Autism in Toddlers, Revised (M-CHAT-R) Screenings Between Children Born Before and During the Pandemic Across the Electronic Health Record (COMBO-EHR) and Research (COMBO-RSCH) Cohorts

Variable	B (SE)	aOR (95% CI)	*P* value
Born before (n = 442) and during the pandemic (n = 1222) groups in COMBO-EHR			
Pandemic birth^a^^,^^b^	−0.29 (0.19)	0.75 (0.52 to 1.08)	.12
Age at M-CHAT administration, mo	−0.04 (0.02)	0.96 (0.93 to 1.00)	.05
Gestational age, wk	−0.10 (0.03)	0.90 (0.86 to 0.95)	<.001
Maternal age at delivery, y	−0.01 (0.01)	0.99 (0.97 to 1.01)	.16
Infant sex assigned at birth (male)	0.61 (0.12)	1.84 (1.45 to 2.35)	<.001
Insurance (Medicaid)	0.75 (0.20)	2.11 (1.45 to 3.14)	<.001
Maternal race			
American Indian or Alaska Native^c^	−0.30 (1.12)	0.74 (0.04 to 4.72)	.79
Asian	0.08 (0.44)	1.08 (0.43 to 2.45)	.86
Black or African American	0.34 (0.20)	1.41 (0.95 to 2.09)	.09
White	0.09 (0.18)	1.10 (0.77 to 1.56)	.61
Declined	0.29 (0.23)	1.33 (0.83 to 2.10)	.22
Unknown	0.26 (0.31)	1.29 (0.71 to 2.35)	.41
Maternal ethnicity			
Non-Hispanic or Latino or Spanish origin^d^	−0.29 (0.19)	0.75 (0.51 to 1.09)	.13
Declined	−0.32 (0.33)	0.72 (0.37 to 1.35)	.32
Unknown	−0.66 (0.28)	0.51 (0.29 to 0.89)	.02
Born before (n = 74) and during the pandemic (n = 311) groups in COMBO-RSCH			
Pandemic birth^b^^,^^e^	0.34 (0.40)	1.40 (0.66 to 3.23)	.40
Age at M-CHAT administration, mo	−0.41 (0.24)	0.66 (0.40 to 1.00)	.08
Gestational age, wk	−0.21 (0.09)	0.81 (0.67 to 0.97)	.02
Maternal age at delivery, y	−0.07 (0.03)	0.93 (0.87 to 0.99)	.02
Infant sex assigned at birth (male)	0.77 (0.32)	2.16 (1.17 to 4.16)	.02
Insurance (Medicaid)	0.60 (0.38)	1.82 (0.87 to 3.90)	.12
Maternal race			
American Indian or Alaska Native^c^	1.24 (1.39)	3.47 (0.13 to 48.23)	.37
Asian	1.30 (0.80)	3.67 (0.66 to 16.68)	.11
Black or African American	0.54 (0.54)	1.71 (0.57 to 4.88)	.32
Native Hawaiian or other Pacific Islander	15.71 (1455.40)	NA^f^	.99
White	0.40 (0.41)	1.49 (0.67 to 3.34)	.33
Declined	−0.59 (0.59)	0.56 (0.16 to 1.67)	.32
Maternal ethnicity			
Not Hispanic or Latino or Spanish origin^d^	−0.52 (0.43)	0.59 (0.26 to 1.38)	.22
Declined	0.41 (0.57)	1.50 (0.48 to 4.60)	.47
Unknown	−13.80 (1455.40)	NA^f^	.99

Abbreviations: NA, not applicable; aOR, adjusted odds ratio.

^a^ Prevalence of elevated M-CHAT-R score in COMBO-EHR group born before the pandemic (reference) is 22.6%.

^b^ Pandemic birth is defined as being born on or after March 1, 2020.

^c^ Reference group for maternal race categories is other combinations not described.

^d^ Reference group for maternal ethnicity is Hispanic or Latino.

^e^ Prevalence of elevated M-CHAT-R score in COMBO-RSCH group born before the pandemic (reference) is 13.5%.

^f^ The true value cannot be determined owing to the small sample size.

### Prenatal Pandemic Exposure and M-CHAT-R Positive Screening: COMBO-RSCH Cohort

There was no difference in the proportion of M-CHAT-R positive screenings between children born before the pandemic and those born during the pandemic (χ^2^_1_ = 0.32; *P* = .57). Specifically, 10 children (13.5%) born before the pandemic and 53 children (17.0%) born during the pandemic screened positive (Figure, B). The lack of an association was maintained in a fully adjusted model (aOR, 1.40; 95% CI, 0.66-3.23; *P* = .40) (Table 3) and a sensitivity analysis excluding children born in March 2020 (eTable 6 in Supplement 1). No associations were observed in adjusted subgroup analyses of preterm, full-term, male, female, Hispanic, not Hispanic, Medicaid-insured, and commercially insured children (eTable 7, eTable 8, eTable 9, and eTable 10 in Supplement 1).

### Prenatal SARS-CoV-2 Exposure and M-CHAT-R Positive Screenings: COMBO-EHR Cohort

Opposite to our hypothesis, there was an unanticipated significant association between prenatal SARS-CoV-2 exposure and lower rates of M-CHAT-R positive screenings (χ^2^_1_ = 8.28; *P* = .004). Specifically, 239 unexposed children (24.0%) vs 16 exposed children (12.3%) screened positive (Figure, C). This association remained significant in a fully adjusted model (aOR, 0.40; 95% CI, 0.22-0.68; *P* = .001) (Table 4) and persisted in sensitivity analyses excluding cases with elevated potential for misclassification of SARS-CoV-2 exposure due to missing serological testing (427 cases; aOR, 0.43; 95% CI, 0.22-0.81; *P* = .01) (eTable 11 in Supplement 1). The association remained significant among full-term children (982 children; aOR, 0.45; 95% CI, 0.24-0.79; *P* = .01), but did not reach statistical significance among preterm children (145 children; aOR, 0.16; 95% CI, 0.01-0.84; *P* = .07) (eTable 12 in Supplement 1). The association was significant in female (555 children; aOR, 0.30; 95% CI, 0.10-0.71; *P* = .01), male (572 children; aOR, 0.48; 95% CI, 0.23-0.93; *P* = .04), Hispanic (774 children; aOR, 0.44; 95% CI, 0.23-0.77; *P* = .01), and Medicaid-insured (890 children; aOR, 0.39; 95% CI, 0.21-0.68; *P* = .002) children, but not in non-Hispanic or commercially insured children (eTable 13, eTable 14, and eTable 15 in Supplement 1).

**Table 4.** Comparison of Rates of Positive Modified Checklist for Autism in Toddlers, Revised (M-CHAT-R) Screenings Between SARS-CoV-2 Exposed and Unexposed Children Across the Electronic Health Record (COMBO-EHR) and Research (COMBO-RSCH) Cohorts

Variable	B (SE)	aOR (95% CI)	*P* value
SARS-CoV-2 unexposed (n = 997) and exposed (n = 130) groups in COMBO-EHR			
SARS-CoV-2 infection in pregnancy^a^	−0.91 (0.28)	0.40 (0.22 to 0.68)	.001
Age at M-CHAT administration, mo	−0.04 (0.02)	0.96 (0.92 to 1.01)	.09
Gestational age, wk	−0.13 (0.04)	0.88 (0.82 to 0.94)	<.001
Maternal age at delivery	−0.01 (0.01)	0.99 (0.96 to 1.01)	.34
Infant sex assigned at birth (male)	0.54 (0.15)	1.71 (1.28 to 2.30)	<.001
Insurance (Medicaid)	0.56 (0.25)	1.75 (1.09 to 2.87)	.02
Maternal race			
American Indian or Alaska Native^b^	0.37 (1.27)	1.45 (0.06 to 14.64)	.77
Asian	−0.13 (0.59)	0.88 (0.24 to 2.58)	.83
Black or African American	0.26 (0.24)	1.29 (0.80 to 2.08)	.29
White	−0.05 (0.22)	0.95 (0.61 to 1.45)	.80
Declined	0.46 (0.26)	1.58 (0.95 to 2.60)	.07
Maternal ethnicity			
Not Hispanic or Latino or Spanish origin^c^	−0.39 (0.24)	0.68 (0.42 to 1.08)	.11
Declined	−0.55 (0.36)	0.58 (0.28 to 1.14)	.12
SARS-CoV-2 unexposed (n = 201) and exposed (n = 101) groups in COMBO-RSCH			
SARS-CoV-2 infection in pregnancy^d^	−0.67 (0.37)	0.51 (0.24 to 1.04)	.07
Age at M-CHAT administration, mo	−0.37 (0.25)	0.69 (0.39 to 1.05)	.15
Gestational age, wk	−0.20 (0.12)	0.82 (0.64 to 1.03)	.09
Maternal age at delivery, y	−0.07 (0.03)	0.93 (0.87 to 0.99)	.03
Infant sex assigned at birth (male)	0.65 (0.35)	0.91 (0.98 to 3.88)	.06
Insurance (Medicaid)	0.56 (0.42)	1.74 (0.78 to 4.05)	.18
Maternal race			
American Indian or Alaska Native^b^	1.06 (1.44)	2.88 (0.10 to 44.93)	.46
Asian	1.24 (0.84)	3.47 (0.59 to 17.29)	.14
Black or African American	0.31 (0.66)	1.37 (0.35 to 4.79)	.64
Native Hawaiian/other Pacific Islander	15.38 (1455.40)	NA^e^	.99
White	0.45 (0.45)	1.57 (0.65 to 3.85)	.31
Declined	−1.10 (0.71)	0.33 (0.08 to 1.23)	.12
Maternal ethnicity			
Not Hispanic or Latino or Spanish origin^c^	−0.76 (0.47)	0.47 (0.18 to 1.17)	.11
Declined	0.51 (0.66)	1.67 (0.45 to 6.24)	.44
Unknown	−13.67 (1455.40)	NA^e^	.99

Abbreviations: NA, not applicable; aOR, adjusted odds ratio.

^a^ Prevalence of elevated M-CHAT-R score in COMBO-EHR SARS-CoV-2 unexposed (reference) group is 24.0%.

^b^ Reference group for maternal race categories is other combinations not described.

^c^ Reference group for maternal ethnicity is Hispanic or Latino.

^d^ Prevalence of elevated M-CHAT-R score in COMBO-RSCH SARS-CoV-2 unexposed (reference) group is 19.9%.

^e^ The true value cannot be determined owing to the small sample size.

### Prenatal SARS-CoV-2 Exposure and M-CHAT-R Positive Screenings: COMBO-RSCH Cohort

A similar, although nonsignificant, finding of lower rates of M-CHAT-R positive screenings in children with prenatal SARS-CoV-2 exposure was observed in the COMBO-RSCH cohort (40 unexposed children [19.9%] vs 13 exposed children [12.9%]; χ^2^_1_ = 0.32; *P* = .57) (Figure, D). No association was found in a fully adjusted model (aOR, 0.51; 95% CI, 0.24-1.04; *P* = .07). The association was significant for Medicaid-insured children, but not for preterm, full-term, male, female, Hispanic, not Hispanic, or commercially insured children (eTable 16, eTable 17, eTable 18, and eTable 19 in Supplement 1).

## Discussion

Considering conflicting evidence on associations between COVID-19 and infant neurodevelopment, continued long-term monitoring of children born during the COVID-19 pandemic is important for public health and educational policy. In this cohort study, we evaluated neurodevelopmental risk of children born before and during the COVID-19 pandemic and those with and without prenatal SARS-CoV-2 exposure using the M-CHAT-R, a screening tool widely used for clinical and research purposes, in a demographically diverse sample in NYC. We found no increase in positive screening rates for autism for children born during the pandemic compared with children born before the pandemic. Surprisingly, we found that prenatal maternal SARS-CoV-2 infection was associated with lower rates of positive autism screenings.

Overall, our sample had higher M-CHAT-R positivity rates compared with other samples (22%-23% compared with 9% in a general population).^27^ Participants in our analysis primarily resided in an urban city, where the prevalence of autism diagnoses is higher.^28^ Our cohort had a high percentage of Hispanic participants, a population found to have 20% to 25% M-CHAT-R positivity rates.^29,30,31,32,33^ Furthermore, validation of the Spanish version of the M-CHAT-R was conducted in Spain, which may differ culturally from our study sample.^34^ Our cohort is also uniquely enriched with children of low SES, which also has been associated with higher positivity rates.^26^ However, subgroup analyses did not reveal compelling evidence for our main results differing by any of these known risk factors.

Reassuringly, maternal SARS-CoV-2 exposure during pregnancy was not associated with increased risk for screening positive on the M-CHAT-R. Research suggests that SARS-CoV-2 may cause MIA, with moderate but temporary changes in cytokine levels during pregnancy^35^ that could be associated with child neurodevelopmental risk.^10^ Our findings align with those of other reports^4,5,7,12^ showing no or limited associations between prenatal SARS-CoV-2 exposure and child neurodevelopment. Many of these studies, however, including ours, primarily include participants with mild illness. Additional exploration of the relationship between severity of infection and neurodevelopment is needed.

For many, the social and economic impact of the COVID-19 pandemic may continue to affect developmental trajectories of children born during the pandemic.^36^ There may also be intergenerational consequences that could worsen existing inequalities.^37^ Furthermore, data suggest that the impact of prenatal exposures may not manifest until higher cognitive functions begin to emerge and mature.^38^ The COVID-19 pandemic has resulted in increased mental health needs among pregnant and postpartum individuals,^39^ and higher levels of the stress hormone cortisol during pregnancy is associated with lower offspring educational attainment.^40^ At the same time, the pandemic catalyzed a number of changes in the education system.^41^

### Limitations

This study has limitations. Children with prenatal SARS-CoV-2 exposure had M-CHAT-R positivity rates close to the expected rate in a general population, whereas unexposed children had higher-than-expected positive screening rates.^42^ Unmeasured differences between exposed and unexposed groups in conjunction with the subjective nature of the M-CHAT-R likely contribute to the unexpected direction of this association. Data suggest that maternal dispositional variables relate to reporting of their child’s behavior.^43^ Mothers who experienced greater stress and vigilance toward the prevention of SARS-CoV-2 may be less likely to become infected and more likely to monitor and report concerning behaviors through the M-CHAT-R.^44,45^ Other confounding variables unaccounted for include information about maternal conditions (eg, hypertension and gestational diabetes), familial risk, and other factors known to confer greater likelihood for neurodevelopmental disorders. Although it is beyond the scope of this analysis, further studies should examine the interactive effects of social determinants of health and prenatal SARS-CoV-2 infection on child neurodevelopment given the health inequalities experienced by marginalized communities during the pandemic. Large-scale collaborative efforts across multiple medical systems will be needed to examine these impacts with adequate statistical power.

Additionally, the unexpected direction of our significant findings may be due to limitations of the M-CHAT-R. Most notably, we focus on autism risk screening, without follow-up of actual diagnoses. Furthermore, although groups of children born before the pandemic without prenatal exposure to the pandemic milieu were included, all children were assessed during the pandemic, possibly affecting maternal report, especially for M-CHAT-R screenings obtained during the early phase of the pandemic when clinical practice was most impacted by quarantine. This may limit the interpretability of null findings of prenatal pandemic exposure. Note that this limitation does not apply to analyses of prenatal SARS-CoV-2 exposure given that screenings for the exposed and unexposed groups were obtained at comparable times during the pandemic.

## Conclusions

Our findings suggest that neither prenatal exposure to maternal SARS-CoV-2 infection nor prenatal exposure to the pandemic milieu is associated with likelihood of positive screening results for autism. Continued monitoring of this generation is important to develop targeted and appropriate policies in education and welfare.

## Notes

Supplement 1. **eMethods 1. **Estimated Misclassification of Maternal SARS-CoV-2 Status During Pregnancy **eMethods 2. **Comparing the COMBO-RSCH Cohort and the COMBO-EHR Cohort **eFigure 1. **Flowchart of COMBO-EHR and COMBO-RSCH Cohorts **eFigure 2. **SARS-CoV-2 Status Determination **eTable 1. **Sensitivity Analyses of M-CHAT-R Positive Screenings Excluding Cases Born in March 2020 (n=1585) (COMBO-EHR) **eTable 2. **Birth Timing and M-CHAT-R Positive Screenings in Preterm and Full-Term Subgroups (COMBO-EHR) **eTable 3. **Birth Timing and M-CHAT-R Positive Screenings in Female and Male Subgroups (COMBO-EHR) **eTable 4. **Birth Timing and M-CHAT-R Positive Screenings Among Mothers With Hispanic/Latino and Non-Hispanic/Latino Ethnicity (COMBO-EHR) **eTable 5. **Birth Timing and M-CHAT-R Positive Screenings in Commercial and Medicaid Insured Subgroups (COMBO-EHR) **eTable 6. **Sensitivity Analyses of M-CHAT-R Positive Screenings Excluding Cases Born in March 2020 (n=380) (COMBO-RSCH) **eTable 7. **Birth Timing and M-CHAT-R Positive Screenings in Preterm and Full-Term Subgroup (COMBO-RSCH) **eTable 8. **Birth Timing and M-CHAT-R Positive Screenings in Female and Male Subgroups (COMBO-RSCH) **eTable 9. **Birth Timing and M-CHAT-R Positive Screenings Among Mothers With Hispanic/Latino and Non-Hispanic/Latino Ethnicity (COMBO-RSCH) **eTable 10. **Birth Timing and M-CHAT-R Positive Screenings in Commercial and Medicaid Insured Subgroups (COMBO-RSCH) **eTable 11. **Sensitivity Analyses of M-CHAT-R Positive Screenings Excluding Cases Without Serology Testing (n=427) (COMBO-EHR) **eTable 12. **SARS-CoV-2 Exposure and M-CHAT-R Positive Screenings in Preterm and Full-Term Subgroup (COMBO-EHR) **eTable 13. **SARS-CoV-2 and MCHAT-R Positive Screenings in Female and Male Subgroups (COMBO-EHR) **eTable 14. **SARS-CoV-2 Exposure and M-CHAT-R Positive Screenings in Ethnicity Subgroups (Hispanic/Latino or Not Hispanic/Latino; COMBO-EHR) **eTable 15. **SARS-CoV-2 Exposure and M-CHAT-R Positive Screenings in Commercial and Medicaid Insured Subgroups (COMBO-EHR) **eTable 16. **SARS-CoV-2 Exposure and M-CHAT-R Positive Screenings in Preterm and Full-Term Subgroup (COMBO-RSCH) **eTable 17. **SARS-CoV-2 Exposure and M-CHAT-R Positive Screenings in Female and Male Subgroups (COMBO-RSCH) **eTable 18. **SARS-CoV-2 Exposure and M-CHAT-R Positive Screenings Among Mothers With Hispanic/Latino and Non-Hispanic/Latino Ethnicity (COMBO-RSCH) **eTable 19. **SARS-CoV-2 Exposure and M-CHAT-R Positive Screenings in Commercial and Medicaid Insured Subgroups (COMBO-RSCH)

Supplement 2.
Data Sharing Statement
