# Supplementary material for: Positive Autism Screening Rates in Toddlers Born During the COVID-19 Pandemic
Source: JAMA Netw Open. 2024 Sep 23;7(9):e2435005. doi: 10.1001/jamanetworkopen.2024.35005 (PMC11420691; doi:10.1001/jamanetworkopen.2024.35005)
Supplement: Supplement 1. — eMethods 1. Estimated Misclassification of Maternal SARS-CoV-2 Status During Pregnancy eMethods 2. Comparing the COMBO-RSCH Cohort and the COMBO-EHR Cohort eFigure 1. Flowchart of COMBO-EHR and COMBO-RSCH Cohorts eFigure 2. SARS-CoV-2 Status Determination eTable 1. Sensitivity Analyses of M-CHAT-R Positive Screenings Excluding Cases Born in March 2020 (n=1585) (COMBO-EHR) eTable 2. Birth Timing and M-CHAT-R Positive Screenings in Preterm and Full-Term Subgroups (COMBO-EHR) eTable 3. Birth Timing and M-CHAT-R Positive Screenings in Female and Male Subgroups (COMBO-EHR) eTable 4. Birth Timing and M-CHAT-R Positive Screenings Among Mothers With Hispanic/Latino and Non-Hispanic/Latino Ethnicity (COMBO-EHR) eTable 5. Birth Timing and M-CHAT-R Positive Screenings in Commercial and Medicaid Insured Subgroups (COMBO-EHR) eTable 6. Sensitivity Analyses of M-CHAT-R Positive Screenings Excluding Cases Born in March 2020 (n=380) (COMBO-RSCH) eTable 7. Birth Timing and M-CHAT-R Positive Screenings in Preterm and Full-Term Subgroup (COMBO-RSCH) eTable 8. Birth Timing and M-CHAT-R Positive Screenings in Female and Male Subgroups (COMBO-RSCH) eTable 9. Birth Timing and M-CHAT-R Positive Screenings Among Mothers With Hispanic/Latino and Non-Hispanic/Latino Ethnicity (COMBO-RSCH) eTable 10. Birth Timing and M-CHAT-R Positive Screenings in Commercial and Medicaid Insured Subgroups (COMBO-RSCH) eTable 11. Sensitivity Analyses of M-CHAT-R Positive Screenings Excluding Cases Without Serology Testing (n=427) (COMBO-EHR) eTable 12. SARS-CoV-2 Exposure and M-CHAT-R Positive Screenings in Preterm and Full-Term Subgroup (COMBO-EHR) eTable 13. SARS-CoV-2 and MCHAT-R Positive Screenings in Female and Male Subgroups (COMBO-EHR) eTable 14. SARS-CoV-2 Exposure and M-CHAT-R Positive Screenings in Ethnicity Subgroups (Hispanic/Latino or Not Hispanic/Latino; COMBO-EHR) eTable 15. SARS-CoV-2 Exposure and M-CHAT-R Positive Screenings in Commercial and Medicaid Insured Subgroups (COMBO-EHR) eTable 16. S [file jamanetwopen-e2435005-s001.pdf]

Supplemental Online Content

Firestein MR, Manessis A, Warmingham J, et al. Positive autism screening rates in toddlers born during the COVID-19 pandemic. *JAMA Netw Open*. 2024;7(9):e2435005. doi:10.1001/jamanetworkopen.2024.35005

eMethods 1. Estimated Misclassification of Maternal SARS-CoV-2 Status During Pregnancy
eMethods 2. Comparing the COMBO-RSCH Cohort and the COMBO-EHR Cohort
eFigure 1. Flowchart of COMBO-EHR and COMBO-RSCH Cohorts
eFigure 2. SARS-CoV-2 Status Determination
eTable 1. Sensitivity Analyses of M-CHAT-R Positive Screenings Excluding Cases Born in March 2020 (n=1585) (COMBO-EHR)
eTable 2. Birth Timing and M-CHAT-R Positive Screenings in Preterm and Full-Term Subgroups (COMBO-EHR)
eTable 3. Birth Timing and M-CHAT-R Positive Screenings in Female and Male Subgroups (COMBO-EHR)
eTable 4. Birth Timing and M-CHAT-R Positive Screenings Among Mothers With Hispanic/Latino and Non-Hispanic/Latino Ethnicity (COMBO-EHR)
eTable 5. Birth Timing and M-CHAT-R Positive Screenings in Commercial and Medicaid Insured Subgroups (COMBO-EHR)
eTable 6. Sensitivity Analyses of M-CHAT-R Positive Screenings Excluding Cases Born in March 2020 (n=380) (COMBO-RSCH)
eTable 7. Birth Timing and M-CHAT-R Positive Screenings in Preterm and Full-Term Subgroup (COMBO-RSCH)
eTable 8. Birth Timing and M-CHAT-R Positive Screenings in Female and Male Subgroups (COMBO-RSCH)
eTable 9. Birth Timing and M-CHAT-R Positive Screenings Among Mothers With Hispanic/Latino and Non-Hispanic/Latino Ethnicity (COMBO-RSCH)
eTable 10. Birth Timing and M-CHAT-R Positive Screenings in Commercial and Medicaid Insured Subgroups (COMBO-RSCH)
eTable 11. Sensitivity Analyses of M-CHAT-R Positive Screenings Excluding Cases Without Serology Testing (n=427) (COMBO-EHR)

© 2024 Firestein MR et al. *JAMA Network Open.*

eTable 12. SARS-CoV-2 Exposure and M-CHAT-R Positive Screenings in Preterm and Full-Term Subgroup (COMBO-EHR)
eTable 13. SARS-CoV-2 and MCHAT-R Positive Screenings in Female and Male Subgroups (COMBO-EHR)
eTable 14. SARS-CoV-2 Exposure and M-CHAT-R Positive Screenings in Ethnicity Subgroups (Hispanic/Latino or Not Hispanic/Latino; COMBO-EHR)
eTable 15. SARS-CoV-2 Exposure and M-CHAT-R Positive Screenings in Commercial and Medicaid Insured Subgroups (COMBO-EHR)
eTable 16. SARS-CoV-2 Exposure and M-CHAT-R Positive Screenings in Preterm and Full-Term Subgroup (COMBO-RSCH)
eTable 17. SARS-CoV-2 Exposure and M-CHAT-R Positive Screenings in Female and Male Subgroups (COMBO-RSCH)
eTable 18. SARS-CoV-2 Exposure and M-CHAT-R Positive Screenings Among Mothers With Hispanic/Latino and Non-Hispanic/Latino Ethnicity (COMBO-RSCH)
eTable 19. SARS-CoV-2 Exposure and M-CHAT-R Positive Screenings in Commercial and Medicaid Insured Subgroups (COMBO-RSCH)

This supplemental material has been provided by the authors to give readers additional information about their work.

© 2024 Firestein MR et al. *JAMA Network Open.*

eMethods 1. Estimated misclassification of maternal SARS-CoV-2 status during pregnancy

The COMBO Initiative utilizes NYP's Clinical Data Warehouse to automatically extract data, including maternal SARS-CoV-2 test results by PCR and serology, on all delivering mothers and their infants every two weeks. After receiving the data through automated EHR extraction, a detailed chart review was conducted for each SARS-CoV-2 exposed mother/infant dyad to determine symptom severity and timing relative to the pregnancy (e.g., pre-pregnancy, first, second, or third trimester, after delivery). This chart review was primarily conducted by pediatricians and obstetricians providing patient care during the pandemic, or research assistants trained by these clinicians. For women identified as exposed based on serology only, the chart was reviewed for outside records scanned into our EHR system, in person and telehealth visits, and all other notes between onset of the pandemic (early March 2020) and birth. False positive SARS-CoV-2 results by both PCR and serology are extremely rare and most of our patients had either multiple positive tests or experienced symptoms. Therefore, the potential for misclassifying unexposed mothers into the exposed group is very low and was not considered in our exploration of effects of misclassification. For mothers classified as being unexposed to SARS-CoV-2 during pregnancy, there were two sources of potential misclassification: asymptomatic disease and nonseroconversion. The asymptomatic disease in our population has been estimated to be approximately 30%. The most conservative estimate of misclassification of exposed dyads into the unexposed group prior to 7/20/2020 (when universal testing by serology was initiated) is therefore 30% of the 15% of women expected to have COVID-19 disease during pregnancy, which results in 4.5%. After 7/20/2020, misclassification would occur most likely due to asymptomatic disease in nonseroconverters. Therefore, the most conservative rate of misclassification would be 15% of the 4.5% of asymptomatic women or 0.67% of all women initially screened into the unexposed group.

© 2024 Firestein MR et al. *JAMA Network Open.*

eMethods 2. Comparing the COMBO-RSCH Cohort and the COMBO-EHR Cohort

We evaluated potential differences between children in the COMBO-EHR cohort and children in the COMBO-RSCH cohort to further understand the observed discordant results. Independent samples T-tests revealed that children in the COMBO-EHR cohort were significantly older at the time of the M-CHAT-R assessment compared to children in the COMBO-RSCH cohort ($t = 30.24$, $p < 0.01$). Overall, children in the COMBO-RSCH cohort had lower M-CHAT-R scores than children in the COMBO-EHR cohort ($t = 5.5245$, $p < 0.01$) and Mann Whitney U test confirmed significantly lower proportion of children in the COMBO-RSCH cohort screened positive on the M-CHAT-R compared to children in the COMBO-EHR cohort ($W = 276830$, $p < 0.01$).

© 2024 Firestein MR et al. *JAMA Network Open.*

eFigure 1. Flowchart of COMBO-EHR and COMBO-RSCH Cohorts

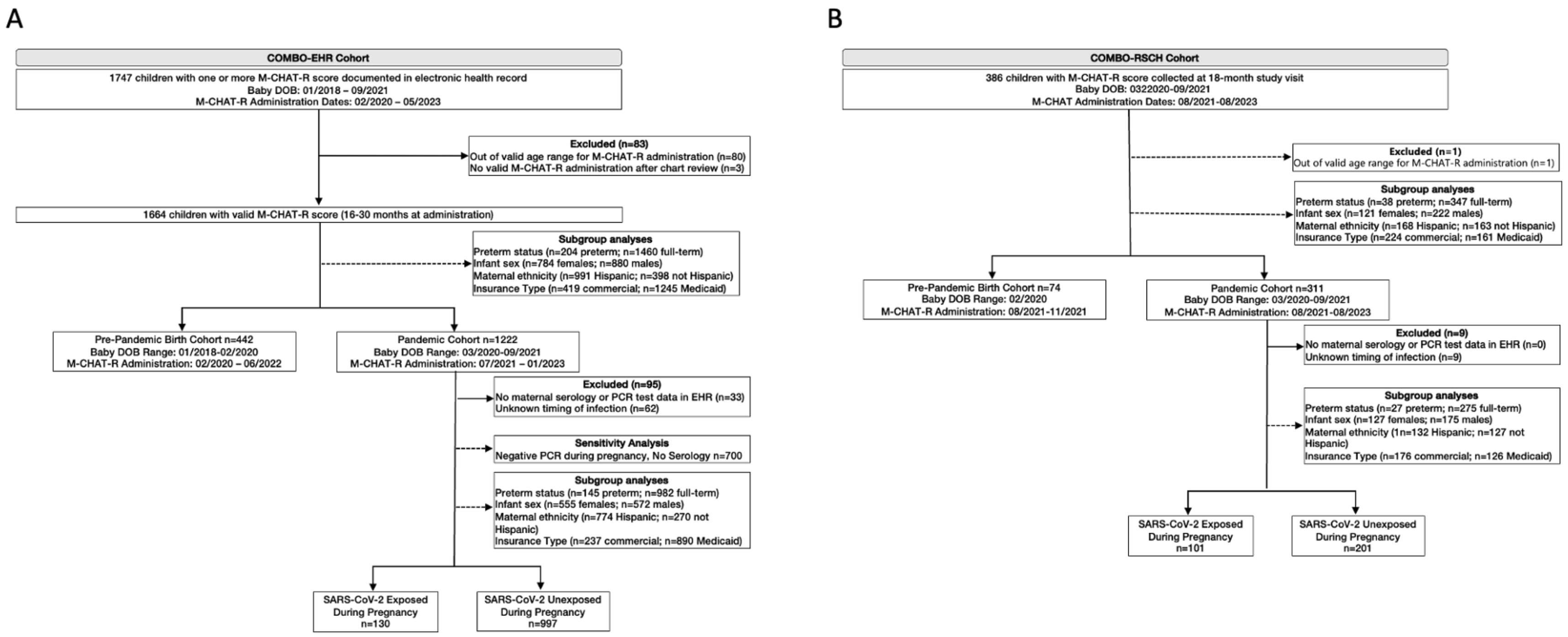

© 2024 Firestein MR et al. *JAMA Network Open.*

eFigure 2. SARS-CoV-2 status determination

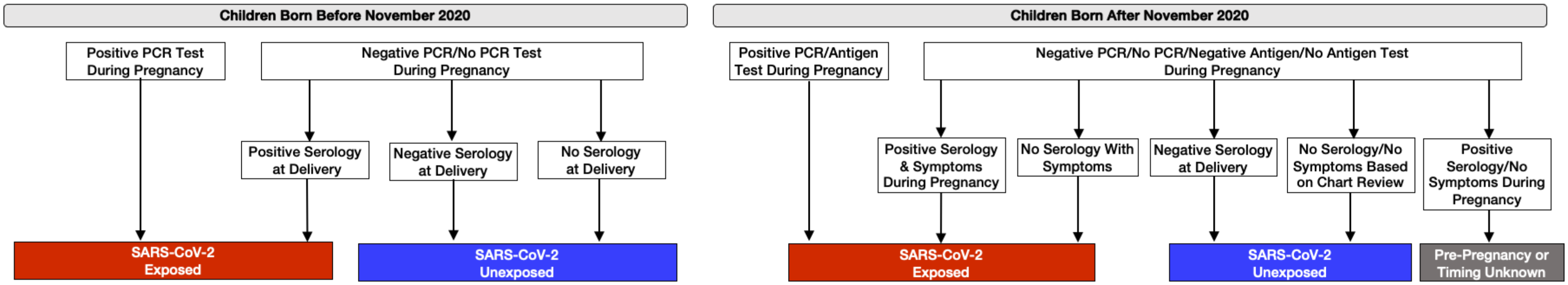

© 2024 Firestein MR et al. *JAMA Network Open.*

eTable 1. Sensitivity Analyses of M-CHAT-R Positive Screenings Excluding Cases Born in March 2020 (n=1585[a]) (*COMBO-EHR)*

Predictor	B	SE	Odds Ratio 95% CI	P-value
Pandemic Birth (*Born on or after 04/01/2020*)	-0.25	0.19	0.78<br>0.54 – 1.14	0.19
Age at M-CHAT-R administration (months)	-0.03	0.02	0.97<br>0.94 – 1.01	0.16
Gestational age (weeks)	-0.10	0.03	0.91<br>0.86 – 0.96	<0.001
Maternal age at delivery	-0.01	0.01	0.99<br>0.97 – 1.01	0.21
Infant sex assigned at birth (Male)	0.64	0.13	1.90<br>1.48 – 2.44	<0.001
Insurance (Medicaid)	0.76	0.21	2.15<br>1.45 – 3.24	<0.001
Maternal Race – American Indian or Alaska Native	0.10	1.15	1.10<br>0.05 – 7.85	0.93
Maternal Race – Asian	0.17	0.44	1.18<br>0.47 – 2.70	0.71
Maternal Race – Black or African American	0.40	0.21	1.49<br>0.99 – 2.22	0.05
Maternal Race – Declined	0.29	0.24	1.34<br>0.82 – 2.14	0.23
Maternal Race – Unknown	0.27	0.31	1.31<br>0.71 – 2.40	0.38
Maternal Race – White	0.11	0.19	1.12<br>0.77 – 1.62	0.54
Maternal Ethnicity – Declined	-0.30	0.33	0.74<br>0.38 – 1.40	0.37
Maternal Ethnicity – Not Hispanic or Latino or Spanish Origin	-0.33	0.20	0.72<br>0.49 - 1.05	0.09
Maternal Ethnicity – Unknown	-0.68	0.28	0.51<br>0.29 - 0.88	0.02

[a] Prevalence of elevated M-CHAT-R score in COMBO-EHR in pre-pandemic birth group is 0.23

© 2024 Firestein MR et al. *JAMA Network Open.*

eTable 2. Birth Timing and M-CHAT-R Positive Screenings in Preterm and Full-Term Subgroups (COMBO-EHR)				
**Predictor**	**B**	**SE**	**Odds Ratio 95% CI**	**P-value**
Preterm Subgroup (n=204[a])				
Pandemic Birth (*Born on or after 03/01/2020*)	0.06	0.58	1.06<br>0.35 - 3.42	0.92
Age at M-CHAT-R administration (months)	0.04	0.05	1.04<br>0.94 - 1.15	0.43
Maternal age at delivery	-0.04	0.03	0.96<br>0.91 - 1.02	0.18
Infant sex assigned at birth (Male)	0.52	0.36	1.68<br>0.84 -3.44	0.15
Insurance (Medicaid)	0.45	0.54	1.58<br>0.56 - 4.78	0.40
Maternal Race – American Indian or Alaska Native	1.10	1.49	2.99<br>0.11 -84.50	0.46
Maternal Race – Asian	-0.68	1.23	0.51<br>0.02 - 4.24	0.58
Maternal Race – Black or African American	0.36	0.57	1.44<br>0.46 -4.36	0.52
Maternal Race – Declined	-1.03	0.77	0.35<br>0.06 - 1.42	0.18
Maternal Race – Unknown	-0.33	1.14	0.72<br>0.06 - 6.53	0.77
Maternal Race – White	-0.02	0.48	0.98<br>0.38 - 2.47	0.97
Maternal Ethnicity – Declined	0.25	0.99	1.29<br>0.14 – 8.65	0.80
Maternal Ethnicity – Not Hispanic or Latino	0.06	0.54	1.06<br>0.37 - 3.06	0.91
Maternal Ethnicity - Unknown	-1.01	1.08	0.37<br>0.03 – 2.82	0.35
Full-Term Subgroup (n=1460[b])				
Pandemic Birth (*Born on or after 03/01/2020*)	-0.32	0.20	0.72<br>0.49 - 1.07	0.11
Age at M-CHAT-R administration (months)	-0.05	0.02	0.95<br>0.92 - 0.99	0.02
Maternal age at delivery	-0.01	0.01	0.99<br>0.97 - 1.02	0.54
Infant sex assigned at birth (Male)	0.64	0.13	1.89<br>1.46 - 2.46	<.001

© 2024 Firestein MR et al. *JAMA Network Open.*

Predictor	B	SE	Odds Ratio<br>95% CI	P-value
Insurance (Medicaid)	0.79	0.21	2.21<br>1.36 - 3.40	<.001
Maternal Race – American Indian or Alaska Native[c]	-12.60	346.59	NA	0.97
Maternal Race – Asian	0.19	0.47	1.21<br>0.45 - 2.93	0.69
Maternal Race – Black or African American	0.34	0.22	1.41<br>0.92 - 2.15	0.11
Maternal Race – Declined	0.44	0.25	1.56<br>0.95 - 2.52	0.08
Maternal Race – Unknown	0.34	0.32	1.41<br>0.75 - 2.63	0.28
Maternal Race – White	0.13	0.20	1.14<br>0.77 - 1.67	0.50
Maternal Ethnicity - Declined	-0.40	0.35	0.67<br>0.33 – 1.31	0.26
Maternal Ethnicity – Not Hispanic or Latino	-0.37	0.21	0.69<br>0.46 – 1.04	0.08
Maternal Ethnicity – Unknown	-0.57	0.29	0.56<br>0.32 – 0.99	0.05

[a] Prevalence of elevated M-CHAT-R score in COMBO-EHR Preterm pre-pandemic birth group is 0.21
[b] Prevalence of elevated M-CHAT-R score in COMBO-EHR Full-term pre-pandemic birth group is 0.22
[c] 95% CI was not able to be estimated due to small sample size.

© 2024 Firestein MR et al. *JAMA Network Open.*

eTable 3. Birth Timing and M-CHAT-R Positive Screenings in Female and Male Subgroups (COMBO-EHR)

Predictor	B	SE	Odds Ratio 95% CI	P-value
Females (n=784[a])				
Pandemic Birth (*Born on or after 03/01/2020*)	-0.35	0.31	0.71<br>0.39 - 1.32	0.26
Age at M-CHAT-R administration (months)	-0.047	0.03	0.95<br>0.90 - 1.01	0.12
Maternal age at delivery	0.0087	0.017	1.01<br>0.98 – 1.04	0.61
Insurance (Medicaid)	0.97	0.35	2.63<br>1.37 – 5.35	0.01
Gestational Age	-0.088	0.045	0.92<br>0.84 – 1.00	0.05
Maternal Race – American Indian or Alaska Native[b]	-12.48	487.25	NA	0.98
Maternal Race – Asian	0.17	0.70	1.18<br>0.25 – 4.22	0.81
Maternal Race – Black or African American	0.54	0.31	1.71<br>0.93 – 3.12	0.08
Maternal Race – Declined	0.24	0.36	1.27<br>0.61 – 2.50	0.51
Maternal Race – Unknown	0.42	0.55	1.53<br>0.50 – 4.46	0.44
Maternal Race – White	-0.09	0.28	0.91<br>0.52 – 1.58	0.75
Maternal Ethnicity - Declined	-0.09	0.50	0.91<br>0.33 – 2.32	0.85
Maternal Ethnicity – Not Hispanic or Latino	-0.46	0.32	0.63<br>0.34 – 1.17	0.15
Maternal Ethnicity – Unknown	-1.20	0.56	0.30<br>0.09 – 0.86	0.03
Males (n=880[c])				
Pandemic Birth (*Born on or after 03/01/2020*)	-0.29	0.24	0.75<br>0.47 - 1.21	0.23
Age at M-CHAT-R administration (months)	-0.03	0.02	0.97<br>0.92 – 1.01	0.17
Maternal age at delivery	-0.03	0.01	0.97<br>0.95 – 1.0	0.04
Insurance (Medicaid)	0.65	0.25	1.91<br>1.20 – 3.13	0.008
Gestational Age	-0.11	0.03	0.90<br>0.84 –0.96	0.001

© 2024 Firestein MR et al. *JAMA Network Open.*

Predictor	B	SE	Odds Ratio 95% CI	P-value
Maternal Race – American Indian or Alaska Native	0.11	1.18	1.12<br>0.05 – 8.78	0.93
Maternal Race – Asian	0.06	0.57	1.07<br>0.32 – 3.06	0.91
Maternal Race – Black or African American	0.19	0.27	1.21<br>0.71 – 2.04	0.48
Maternal Race – Declined	0.32	0.31	1.37<br>0.73 – 2.52	0.31
Maternal Race – Unknown	0.20	0.37	1.22<br>0.58 – 2.55	0.59
Maternal Race – White	0.20	0.24	1.22<br>0.76 – 1.95	0.41
Maternal Ethnicity - Declined	-0.48	0.43	0.62<br>0.26 – 1.43	0.27
Maternal Ethnicity – Not Hispanic or Latino	-0.19	0.24	0.83<br>0.51 – 1.34	0.45
Maternal Ethnicity – Unknown	-0.50	0.33	0.60<br>0.31 – 1.16	0.13

[a] Prevalence of elevated M-CHAT-R score in COMBO-EHR pre-pandemic birth group among female children is 0.15
[b] 95% CI was not able to be estimated due to small sample size.
[c] Prevalence of elevated M-CHAT-R score in COMBO-EHR pre-pandemic birth group among male children is 0.28

© 2024 Firestein MR et al. *JAMA Network Open.*

eTable 4. Birth Timing and M-CHAT-R Positive Screenings among mothers with Hispanic/Latino and Non-Hispanic/Latino Ethnicity (COMBO-EHR)

Predictor	B	SE	Odds Ratio 95% CI	P-value
Maternal Ethnicity: Hispanic/Latino (n=991[a])				
Pandemic Birth (*Born on or after 03/01/2020*)	-0.30	0.25	0.74 (0.46 - 1.22)	0.23
Age at M-CHAT-R administration (months)	-0.05	0.02	0.95 (0.91 - 0.99)	0.03
Maternal age at delivery	-0.02	0.01	0.98 (0.95 - 1.00)	0.06
Infant Sex assigned at birth (Male)	0.56	0.15	1.74 (1.30 - 2.35)	<.001
Gestational Age	-0.12	0.04	0.88 (0.82 - 0.95)	<.001
Insurance (Medicaid)	0.21	0.28	1.23 (0.72 - 2.20)	0.46
Maternal Race – American Indian or Alaska Native	0.83	1.43	2.28 (0.09 - 58.90)	0.56
Maternal Race – Black or African American	-0.23	0.29	0.79 (0.44 - 1.37)	0.42
Maternal Race – Declined	0.42	0.26	1.52 (0.91 - 2.50)	0.10
Maternal Race – Unknown	0.50	0.42	1.65 (0.72 - 3.73)	0.23
Maternal Race – White	0.29	0.20	1.34 (0.91 - 1.95)	0.14
Maternal Ethnicity: Not Hispanic or Latino (n=398[c])				
Pandemic Birth (*Born on or after 03/01/2020*)	-0.50	0.35	0.60 (0.31 - 1.20)	0.15
Age at M-CHAT-R administration (months)	-0.02	0.04	0.98 (0.90 – 1.07)	0.65
Maternal age at delivery	0.03	0.02	1.03 (0.98 - 1.08)	0.30
Infant Sex assigned at birth (Male)	0.68	0.29	1.97 (1.12 – 3.52)	0.02
Gestational Age	-0.15	0.06	0.86 (0.77 - 0.97)	0.01
Insurance (Medicaid)	0.91	0.40	2.47 (1.15 – 5.45)	0.02
Maternal Race – American Indian or Alaska Native[b]	-16.30	1885.26	NA	0.99
Asian	-0.55	0.65	0.58	0.40

© 2024 Firestein MR et al. *JAMA Network Open.*

(0.16 – 2.09)

Predictor	B	SE	Odds Ratio 95% CI	P-value
Maternal Race – Black or African American	-0.04	0.47	1.04 (0.42 – 2.76)	0.93
Maternal Race – Declined[b]	-16.36	1110.38	NA	0.99
Maternal Race – Unknown[b]	-16.91	2647.89	NA	0.99
Maternal Race – White	-1.11	0.58	0.33 (0.11 - 1.06)	0.06

[a] Prevalence of elevated M-CHAT-R score in COMBO-EHR pre-pandemic birth group among Hispanic/Latino group is 0.33
[b] 95% CI could not able to be estimated due to small sample size.
[c] Prevalence of elevated M-CHAT-R score in COMBO-EHR pre-pandemic birth group among non-Hispanic/Latino group is 0.18

© 2024 Firestein MR et al. *JAMA Network Open.*

eTable 5. Birth Timing and M-CHAT-R Positive Screenings in Commercial and Medicaid Insured Subgroups (COMBO-EHR)

Predictor	B	SE	Odds Ratio 95% CI	P-value
Medicaid Insurance (n=1245[a])				
Pandemic Birth (*Born on or after 03/01/2020*)	-0.36	0.21	0.59 (0.24 - 1.47)	0.08
Age at M-CHAT-R administration (months)	-0.03	0.02	0.90 (0.81 - 1.00)	0.19
Maternal age at delivery	-0.01	0.01	0.97 (0.92 - 1.03)	0.37
Infant Sex assigned at birth (Male)	0.55	0.13	2.61 (1.36 - 5.26)	<.001
Gestational Age	-0.09	0.03	0.88 (0.77 - 1.02)	0.002
Maternal Race – American Indian or Alaska Native[b]	0.08	1.18	NA	0.95
Maternal Race – Asian	-0.61	1.11	0.58 (0.16 - 2.05)	0.58
Maternal Race – Black or African American	0.25	0.22	0.60 (0.18-1.89)	0.26
Maternal Race – Declined	0.35	0.25	0.61 (0.16 - 2.21)	0.16
Maternal Race – Unknown	0.02	0.34	1.59 (0.26 - 9.65)	0.96
Maternal Race – White	0.31	0.19	0.29 (0.10 - 0.80)	0.10
Maternal Ethnicity - Declined	-0.36	0.39	0.59 (0.15 - 2.17)	0.35
Maternal Ethnicity – Not Hispanic or Latino	-0.06	0.23	0.54 (0.23 - 1.30)	0.80
Maternal Ethnicity – Unknown	-0.40	0.32	0.18 (0.03 - 0.77)	0.21
Commercial Insurance(n=419[c])				
Pandemic Birth (*Born on or after 03/01/2020*)	-0.53	0.46	0.59 (0.24 - 1.47)	0.25
Age at M-CHAT-R administration (months)	-0.10	0.05	0.90 (0.81 - 1.00)	0.05
Maternal age at delivery	-0.03	0.03	0.97 (0.92 - 1.03)	0.37
Infant Sex assigned at birth (Male)	0.96	0.34	2.61	0.005

© 2024 Firestein MR et al. *JAMA Network Open.*

			(1.36 - 5.26)	
Gestational Age	-0.13	0.07	0.88 (0.77 - 1.02)	0.07
**Predictor**	**B**	**SE**	**Odds Ratio 95% CI**	**P-value**
Maternal Race – American Indian or Alaska Native[b]	-13.39	719.53	NA	0.99
Maternal Race – Asian	-0.55	0.65	0.58 (0.16 - 2.05)	0.40
Maternal Race – Black or African American	-0.51	0.59	0.60 (0.18 - 1.89)	0.39
Maternal Race – Declined	-0.49	0.67	0.61 (0.16 - 2.21)	0.46
Maternal Race – Unknown	0.47	0.91	1.59 (0.26 - 9.65)	0.61
Maternal Race – White	-1.25	0.53	0.29 (0.10 - 0.80)	0.02
Maternal Ethnicity - Declined	-0.53	0.67	0.59 (0.15 - 2.17)	0.43
Maternal Ethnicity – Not Hispanic or Latino	-0.61	0.44	0.55 (0.23 - 1.30)	0.17
Maternal Ethnicity – Unknown	-1.72	0.78	0.18 (0.03 - 0.77)	0.03

[a] Prevalence of elevated M-CHAT-R score in COMBO-EHR pre-pandemic birth group among those insured by Medicaid is 0.31

[b] 95% CI could not be estimated due to small sample size.

[c] Prevalence of elevated M-CHAT-R score in COMBO-EHR pre-pandemic birth group among those with Commercial Insurance is 0.096

© 2024 Firestein MR et al. *JAMA Network Open.*

eTable 6. Sensitivity Analyses of M-CHAT-R Positive Screenings Excluding Cases Born in March 2020 (n=380[a]) (*COMBO-RSCH)*

Predictor	B	SE	Odds Ratio 95% CI	P-value
Pandemic Birth (*Born on or after 04/01/2020*)	0.33	0.40	1.39 (0.65, 3.21)	0.42
Age at M-CHAT administration (months)	-0.35	0.23	0.70 (0.42, 1.05)	0.13
Gestational age (weeks)	-0.20	0.09	0.82 (0.68, 0.98)	0.03
Maternal age at delivery	-0.08	0.03	0.92 (0.86, 0.98)	0.01
Infant sex assigned at birth (Male)	0.89	0.33	2.44 (1.29, 4.84)	0.01
Insurance (Medicaid)	0.52	0.38	1.68 (0.80, 3.62)	0.18
Maternal Race – American Indian or Alaska Native[b]	1.36	1.40	3.91 (0.15, 5.54)	0.33
Maternal Race – Asian	1.32	0.81	3.76 (0.67, 1.73)	0.10
Maternal Race – Black or African American	0.56	0.55	0.76 (0.58, 5.07)	0.31
Maternal Race – Declined	-0.54	0.59	0.58 (0.17, 1.77)	0.35
Maternal Race – Native Hawaiian or Other Pacific Islander	15.67	1455.40	NA (NA)	0.99
Maternal Race – White	0.42	0.42	1.52 (0.66, 3.48)	0.32
Maternal Ethnicity – Declined	0.42	0.57	1.52 (0.48, 4.68)	0.46
Maternal Ethnicity – Not Hispanic or Latino or Spanish Origin	-0.49	0.43	0.61 (0.26, 1.43)	0.26
Maternal Ethnicity – Unknown	-13.64	1455.40	NA (NA)	0.99

[a] Prevalence of elevated M-CHAT score in COMBO-EHR in pre-pandemic birth group is 0.19
[b] Estimate of 95% CI lower limit not able to be estimated due to small sample size.

© 2024 Firestein MR et al. *JAMA Network Open.*

eTable 7. Birth Timing and M-CHAT-R Positive Screenings in Preterm and Full-Term Subgroup (*COMBO-RSCH)*

Predictor	B	SE	Odds Ratio (95% CI)	P-value
Preterm Subgroup (n=38[a])				
Pandemic Birth (*Born on or after 03/01/2020*)	-2.06	1.46	0.13 (<0.01, 1.74)	0.16
Age at M-CHAT-R administration (months)	0.03	0.88	1.03 (0.19, 7.78)	0.97
Maternal age at delivery	0.31	0.17	1.37 (1.02, 2.04)	0.06
Infant Sex assigned at birth (Male)	1.49	1.37	4.44 (0.40, 129.49)	0.28
Insurance (Medicaid)	4.16	1.82	64.38 (3.60, 5616.49)	0.02
Maternal Race – American Indian or Alaska Native[b]	NA	NA	NA	NA
Maternal Race – Asian[b]	NA	NA	NA	NA
Maternal Race – Black or African American [b]	NA	NA	NA	NA
Maternal Race – Declined[b]	NA	NA	NA	NA
Maternal Race – Native Hawaiian or Other Pacific Islander[b]	NA	NA	NA	NA
Maternal Race – White[b]	NA	NA	NA	NA
Maternal Ethnicity – Declined [b]	NA	NA	NA	NA
Maternal Ethnicity – Not Hispanic or Latino or Spanish Origin [b]	NA	NA	NA	NA
Maternal Ethnicity – Unknown [b]	NA	NA	NA	
Term Subgroup (n=347[d])				
Pandemic Birth (*Born on or after 03/01/2020*)	0.76	0.46	2.13 (0.92, 5.68)	0.10
Age at M-CHAT-R administration (months)	-0.25	0.23	0.78 (0.47, 1.13)	0.27
Maternal age at delivery	-0.09	0.03	0.91 (0.85, 0.97)	0.01
Infant Sex assigned at birth (Male)	0.67	0.34	1.95 (1.02, 3.87)	0.05

© 2024 Firestein MR et al. *JAMA Network Open.*

Predictor	B	SE	Odds Ratio (95% CI)	P-value
Insurance (Medicaid)	0.21	0.41	1.23 (0.56, 2.77)	0.61
Maternal Race – American Indian or Alaska Native[c]	-12.86	1027.20	NA	0.99
Maternal Race – Asian	1.18	0.82	3.25 (0.58, 15.20)	0.15
Maternal Race – Black or African American	0.77	0.59	2.16 (0.65, 6.75)	0.19
Maternal Race – Declined	-0.51	0.59	0.20 (0.18, 1.81)	0.39
Maternal Race – Native Hawaiian or Other Pacific Islander[c]	15.65	145.40	NA	0.99
Maternal Race – White	0.45	0.43	1.47 (0.67, 3.66)	0.30
Maternal Ethnicity -- Declined	0.24	0.57	1.27 (0.40, 3.89)	0.48
Maternal Ethnicity – Not Hispanic or Latino or Spanish Origin	-0.70	0.46	0.50 (0.20, 1.22)	0.13
Maternal Ethnicity – Unknown[c]	-13.83	1455.40	NA	0.99

[a] Prevalence of elevated M-CHAT-R score in COMBO-RSCH Preterm unexposed group is 0.50
[b] Due to small sample size, models could not be estimated with race and ethnicity as covariates so they were omitted.
[c] Estimate of 95% CI lower limit not able to be estimated due to small sample size.
[d] Prevalence of elevated M-CHAT-R score in COMBO-RSCH Full term unexposed group is 0.10

© 2024 Firestein MR et al. *JAMA Network Open.*

eTable 8. Birth Timing and M-CHAT-R Positive Screenings in Female and Male Subgroups (*COMBO-RSCH)*

Predictor	B	SE	Odds Ratio (95% CI)	P-value
Females (n=163[a])				
Pandemic Birth (*Born on or after 03/01/2020*)	1.33	0.96	3.77 (0.71, 33.45)	0.16
Age at M-CHAT-R administration (months)	-0.83	0.54	0.44 (0.13, 0.99)0	0.13
Gestational age (weeks)	-0.34	0.17	0.71 (0.49, 1.00)	0.05
Maternal age at delivery	-00.26	0.09	0.77 (0.63, 0.89)	<0.01
Insurance (Medicaid)	0.60	0.80	1.82 (0.39, 9.47)	0.45
Maternal Race – American Indian or Alaska Native[b]	-14.74	2741.17	NA	1.00
Maternal Race – Asian[b]	-14.42	1567.17	NA	0.99
Maternal Race – Black or African American	0.50	0.94	1.65 (0.23, 10.21)	0.59
Maternal Race – Declined	-1.54	1.46	0.21 (0.01, 2.50)	0.29
Maternal Race – White	-0.05	0.88	0.95 (0.16, 5.46)	0.96
Maternal Ethnicity – Declined	0.35	1.52	1.42 (0.05, 27.28)	0.82
Maternal Ethnicity – Not Hispanic or Latino or Spanish Origin	-0.46	0.89	0.63 (0.11, 3.71)	0.60
Maternal Ethnicity – Unknown	-14.91	3956.18	NA	1.00
Males (n=222[c])				
Pandemic Birth (*Born on or after 03/01/2020*)	0.02	0.46	1.02 (0.42, 2.66)	0.97
Age at M-CHAT-R administration (months)	-0.31	0.28	0.74 (0.40, 1.17)	0.28
Gestational age (weeks)	-0.22	0.12	0.80 (0.62, 1.02)	0.09
Maternal age at delivery	-0.03	0.04	0.97 (0.90, 1.04)	0.43

© 2024 Firestein MR et al. *JAMA Network Open.*

Insurance (Medicaid)	0.51	0.45	1.66 (0.49, 4.13)	0.26
**Predictor**	**B**	**SE**	**Odds Ratio (95% CI)**	**P-value**
Maternal Race – American Indian or Alaska Native	16.51	1455.40	NA	0.99
Maternal Race – Asian	1.90	0.92	6.66 (1.01, 40.17)	0.04
Maternal Race – Black or African American	0.64	0.69	1.90 (0.46, 7.17)	0.35
Maternal Race – Declined	-0.16	0.67	0.85 (0.21, 3.06)	0.81
Maternal Race – Native Hawaiian or Other Pacific Islander[b]	16.60	1455.40	NA	0.99
Maternal Race – White	0.68	0.49	0.98 (0.76, 5.30)	0.17
Maternal Ethnicity – Declined	0.50	0.64	1.65 (0.46, 5.87)	0.43
Maternal Ethnicity – Not Hispanic or Latino or Spanish Origin	-0.51	0.51	0.60 (0.22, 1.64)	0.32

[a] Prevalence of elevated M-CHAT-R score in COMBO-RSCH among female children unexposed to SARS-CoV-2 is 0.06
[b] OR and/or 95% CI could not be estimated due to small sample size in this predictor group.
[c] Prevalence of elevated M-CHAT-R score in COMBO-RSCH among male children unexposed to SARS-CoV-2 is 0.20

© 2024 Firestein MR et al. *JAMA Network Open.*

eTable 9. Birth Timing and M-CHAT-R Positive Screenings among mothers with Hispanic/Latino and Non-Hispanic/Latino Ethnicity (COMBO-RSCH)

Predictor	B	SE	Odds Ratio 95% CI	P-value
Maternal Ethnicity: Hispanic/Latino (n=168[a])				
Pandemic Birth (*Born on or after 03/01/2020*)	0.41	0.57	1.51 (0.52, 5.06)	0.47
Age at M-CHAT-R administration (months)	-0.67	0.57	0.51 (0.24, 0.92)	0.05
Maternal age at delivery	-0.09	0.04	0.92 (0.84, 0.99)	0.04
Infant Sex assigned at birth (Male)	0.28	0.44	1.32 (0.56, 3.20)	0.53
Gestational Age	-0.32	0.14	0.73 (0.55, 0.94)	0.02
Insurance (Medicaid)	0.81	0.52	2.25 (0.85, 6.83)	0.12
Maternal Race – American Indian or Alaska Native[b]	1.86	1.63	6.42 (0.19, 220.48)	0.25
Maternal Race – Asian				
Maternal Race – Black or African American	-0.10	0.88	0.91 (0.12, 4.48)	0.91
Maternal Race – Declined	-0.65	0.83	0.52 (0.07, 2.23)	0.43
Maternal Race – Native Hawaiian or Other Pacific Islander[b]	15.74	1455.40	NA	0.99
Maternal Race – White	0.91	0.47	2.49 (0.99, 6.34)	0.05
Maternal Ethnicity: Not Hispanic or Latino (n=163[c])				
Pandemic Birth (*Born on or after 03/01/2020*)	0.05	0.72	1.05 (0.28, 5.10)	0.95
Age at M-CHAT-R administration (months)	-0.66	0.58	0.52 (0.15, 1.42)	0.26
Maternal age at delivery	-0.06	0.06	0.95 (0.73, 1.07)	0.36
Infant Sex assigned at birth (Male)	1.37	0.62	3.92 (1.26, 1.52)	0.03
Gestational Age	-0.22	0.18	0.80 (0.56, 1.14)	0.22
Insurance (Medicaid)	-0.21	0.97	0.81 (0.11, 5.28)	0.83
Maternal Race – American Indian or Alaska Native[b]	-16.06	3956.18	NA	0.99

© 2024 Firestein MR et al. *JAMA Network Open.*

Predictor	B	SE	Odds Ratio 95% CI	P-value
Maternal Race – Asian	-0.40	1.18	0.67 (0.06, 7.55)	0.73
Maternal Race – Black or African American	0.11	1.02	1.12 (0.16, 10.10)	0.91
Maternal Race – Declined	-15.94	1555.59	NA	0.99
Maternal Race – Unknown				
Maternal Race – White	-1.24	0.95	0.29 (0.05, 2.37)	0.19

[a] Prevalence of elevated M-CHAT-R score in COMBO-RSCH pre-pandemic birth group among Hispanic/Latino group is 0.21
[b] Estimate of 95% CI lower limit not able to be estimated due to small sample size.
[c] Prevalence of elevated M-CHAT-R score in COMBO-RSCH pre-pandemic birth group among non-Hispanic/Latino group is 0.09

© 2024 Firestein MR et al. *JAMA Network Open.*

eTable 10. Birth Timing and M-CHAT-R Positive Screenings in Commercial and Medicaid Insured Subgroups (COMBO-RSCH)

Predictor	B	SE	Odds Ratio 95% CI	P-value
Medicaid Insurance (n=161[a])				
Pandemic Birth (*Born on or after 03/01/2020*)	0.33	0.53	0.38 (0.51, 4.20)	0.54
Age at M-CHAT-R administration (months)	-0.29	0.27	0.74 (0.41, 1.16)	0.27
Maternal age at delivery	-0.09	0.04	0.91 (0.84, 0.99)	0.03
Infant Sex assigned at birth (Male)	0.15	0.44	1.16 (0.50, 2.78)	0.73
Gestational Age	-0.30	0.12	0.74 (0.57, 0.94)	0.01
Maternal Race – American Indian or Alaska Native[b]	17.49	2399.54	NA	0.99
Maternal Race – Asian	--	--	--	--
Maternal Race – Black or African American	0.33	0.73	1.39 (0.31, 5.76)	0.65
Maternal Race – Declined	-0.39	0.75	0.68 (0.13, 2.66)	0.60
Maternal Race – Native Hawaiian or Other Pacific Islander[b]	16.98	2399.54	NA	0.99
Maternal Race – White	0.78	0.51	2.17 (0.79, 5.92)	0.13
Maternal Ethnicity - Declined	0.39	0.85	1.48 (0.10, 1.99)	0.65
Maternal Ethnicity – Not Hispanic or Latino	-0.69	0.74	0.50 (0.10, 1.99)	0.35
Maternal Ethnicity – Unknown[b]	-15.47	2399.54	NA	0.99
Commercial Insurance(n=224[c])				
**Predictor**	**B**	**SE**	**Odds Ratio 95% CI**	**P-value**
Pandemic Birth (*Born on or after 03/01/2020*)	0.55	0.69	1.74 (0.51, 8.22)	0.42
Age at M-CHAT-R administration (months)	-0.47	0.49	0.63 (0.21, 1.41)	0.35
Maternal age at delivery	-0.07	0.06	0.93 (0.83, 1.04)	0.20
Infant Sex assigned at birth (Male)	1.29	0.54	3.63 (1.36, 11.61)	0.02

© 2024 Firestein MR et al. *JAMA Network Open.*

Predictor	B	SE	Odds Ratio 95% CI	P-value
Gestational Age	-0.09	0.17	0.91 (0.64, 1.28)	0.59
Maternal Race – American Indian or Alaska Native[b]	-12.88	1023.51	NA	0.99
Maternal Race – Asian	0.58	0.96	1.78 (0.25, 11.68)	0.55
Maternal Race – Black or African American	0.52	0.52	1.68 (0.25, 10.03)	0.57
Maternal Race – Declined	-0.95	0.98	0.39 (0.05, 2.57)	0.34
Maternal Race – White	-0.29	0.71	0.75 (0.19, 3.17)	0.68
Maternal Ethnicity - Declined	0.62	0.85	1.86 (0.34, 10.16)	0.47
Maternal Ethnicity – Not Hispanic or Latino	-0.03	0.65	0.97 (0.28, 3.75)	0.97
Maternal Ethnicity – Unknown				

[a] Prevalence of elevated M-CHAT-R score in COMBO-RSCH pre-pandemic birth group among those insured by Medicaid is 0.23
[b] Estimate of 95% CI lower limit not able to be estimated due to small sample size.
[c] Prevalence of elevated M-CHAT-R score in COMBO-RSCH pre-pandemic birth group among those with Commercial Insurance is 0.07

© 2024 Firestein MR et al. *JAMA Network Open.*

eTable 11. Sensitivity Analyses of M-CHAT-R Positive Screenings Excluding Cases Without Serology Testing (n=427[a]) (*COMBO-EHR)*

Predictor	B	SE	Odds Ratio 95% CI	P-value
SARS-CoV-2 Infection in Pregnancy	-0.84	0.33	0.43 (0.22 – 0.81)	0.01
Age at M-CHAT-R administration (months)	-0.06	0.04	0.94 (0.86 – 1.02)	0.13
Gestational age (weeks)	-0.19	0.05	0.83 (0.75 – 0.92)	<.001
Maternal age at delivery	-0.04	0.02	0.96 (0.91 – 1.00)	0.06
Infant sex assigned at birth (Male)	0.76	0.28	2.13 (1.25 – 3.71)	0.006
Insurance (Medicaid)	-0.21	0.36	0.81 (0.40 – 1.66)	0.56
Maternal Race – American Indian or Alaska Native[b]	-11.95	607.54	NA	0.98
Maternal Race – Asian	-0.004	0.89	1.00 (0.13 – 4.84)	0.99
Maternal Race – Black or African American	0.90	0.41	2.45 (1.08 – 5.52)	0.03
Maternal Race – Declined	0.67	0.48	1.95 (0.74 – 4.92)	0.16
Maternal Race – White	-0.18	0.40	0.83 (0.37 – 1.79)	0.65
Maternal Ethnicity – Declined	-1.03	0.65	0.36 (0.09 – 1.22)	0.12
Maternal Ethnicity – Not Hispanic or Latino or Spanish Origin	-1.05	0.40	0.35 (0.16 - 0.76)	0.009

[a] Prevalence of elevated M-CHAT-R score in SARS-CoV-2 unexposed group is 0.22
[b] 95% CI could not be estimated due to small sample size.

© 2024 Firestein MR et al. *JAMA Network Open.*

eTable 12. SARS-CoV-2 Exposure and M-CHAT-R Positive Screenings in Preterm and Full-Term Subgroup (*COMBO-EHR)*

Predictor	B	SE	Odds Ratio 95% CI	P-value
Preterm Subgroup (n=145[a])				
SARS-CoV-2 Infection in Pregnancy	-1.83	1.00	0.16 (0.01 - 0.84)	0.07
Age at M-CHAT-R administration (months)	0.11	0.07	1.11 (0.97 - 1.28)	0.12
Gestational age (weeks)	-0.43	0.12	0.65 (0.50 - 0.81)	<.001
Maternal age at delivery	-0.05	0.04	0.95 (0.88 - 1.02)	0.16
Infant sex assigned at birth (Male)	0.19	0.46	1.20 (0.49 - 3.02)	0.69
Insurance (Medicaid)	-0.18	0.74	0.84 (0.20 - 3.75)	0.81
Maternal Race – American Indian or Alaska Native	1.44	1.52	4.23 (0.14 - 123.92)	0.34
Maternal Race – Asian	-1.40	1.39	0.25 (0.01 - 3.06)	0.32
Maternal Race – Black or African American	0.57	0.76	1.77 (0.039 - 8.01)	0.45
Maternal Race – Declined	-1.29	0.92	0.28 (0.03 - 1.40)	0.16
Maternal Race – White	-0.66	0.66	0.52 (0.13 - 1.80)	0.32
Maternal Ethnicity – Declined	0.30	1.13	1.35 (0.13 - 12.54)	0.79
Maternal Ethnicity – Not Hispanic or Latino or Spanish Origin	-0.24	0.75	0.78 (0.17 - 3.42)	0.75
Full-Term Subgroup (n=982[b])				
SARS-CoV-2 Infection in Pregnancy	-0.80	0.30	0.45 (0.24 – 0.79)	0.01
Age at M-CHAT-R administration (months)	-0.05	0.02	0.95 (0.90 – 0.99)	0.03
Gestational age (weeks)	-0.12	0.07	0.88 (0.76 – 1.02)	0.10
Maternal age at delivery	-0.004	0.01	1.00 (0.97 – 1.02)	0.77
Infant sex assigned at birth (Male)	0.59	0.16	1.80 (1.31 – 2.47)	<.001
Insurance (Medicaid)	0.58	0.27	1.78 (1.06 – 3.08)	0.03

© 2024 Firestein MR et al. *JAMA Network Open.*

Predictor	B	SE	Odds Ratio 95% CI	P-value
Maternal Race – American Indian or Alaska Native[c]	-10.92	377.83	NA	0.98
Maternal Race – Asian	-0.04	0.68	0.96 (0.21 – 3.25)	0.96
Maternal Race – Black or African American	0.23	0.27	1.26 (0.74 – 2.11)	0.39
Maternal Race – Declined	0.70	0.28	2.02 (1.17 – 3.44)	0.01
Maternal Race – White	-0.01	0.24	0.99 (0.62 – 1.56)	0.96
Maternal Ethnicity – Declined	-0.77	0.39	0.46 (0.21 – 0.97)	0.05
Maternal Ethnicity – Not Hispanic or Latino or Spanish Origin	-0.49	0.27	0.61 (0.36 – 1.02)	0.06

[a] Prevalence of elevated M-CHAT-R scores in COMBO-EHR preterm group unexposed to SARS-CoV-2 is 0.29
[b] Prevalence of elevated M-CHAT-R scores in COMBO-EHR full term group unexposed to SARS-CoV-2 is 0.23
[c] 95% CI could not be estimated due to small sample size in this predictor group.

© 2024 Firestein MR et al. *JAMA Network Open.*

eTable 13. SARS-CoV-2 and MCHAT-R Positive Screenings in Female and Male Subgroups (*COMBO-EHR)*

Predictor	B	SE	Odds Ratio (95% CI)	P-value
Females (n=555[a])				
SARS-CoV-2 Infection in Pregnancy	-1.21	0.49	0.30 (0.10 - 0.71)	0.01
Age at M-CHAT administration (months)	-0.06	0.04	0.95 (0.88 - 1.01)	0.12
Gestational age (weeks)	-0.13	0.06	0.88 (0.79 - 0.99)	0.02
Maternal age at delivery	0.01	0.02	1.01 (0.97 - 1.05)	0.56
Insurance (Medicaid)	0.79	0.41	2.20 (1.01 - 5.19)	0.06
Maternal Race – American Indian or Alaska Native[b]	-12.96	596.80	NA	0.98
Maternal Race – Asian	-0.60	1.11	0.55 (0.03 – 3.37)	0.59
Maternal Race – Black or African American	0.35	0.36	1.42 (0.68 – 2.87)	0.33
Maternal Race – Declined	0.53	0.39	1.69 (0.76 – 3.60)	0.18
Maternal Race – White	-0.10	0.33	0.91 (0.46 – 1.71)	0.77
Maternal Ethnicity – Declined	-0.43	0.56	0.65 (0.20 – 1.86)	0.44
Maternal Ethnicity – Not Hispanic or Latino or Spanish Origin	-0.48	0.39	0.62 (0.28 - 1.31)	0.21
Males (n=572[c])				
SARS-CoV-2 Infection in Pregnancy	-0.73	0.36	0.48 (0.23 – 0.93)	0.04
Age at M-CHAT administration (months)	-0.03	0.03	0.97 (0.92 - 1.03)	0.31
Gestational age (weeks)	-0.14	0.05	0.87 (0.80 - 0.95)	0.002
Maternal age at delivery	-0.03	0.02	0.97 (0.94 - 1.00)	0.09
Insurance (Medicaid)	0.48	0.31	1.62 (0.89 – 3.05)	0.13
Maternal Race – American Indian or Alaska Native	1.57	1.65	4.82 (0.14 – 168.94)	0.34

© 2024 Firestein MR et al. *JAMA Network Open.*

Predictor	B	SE	Odds Ratio (95% CI)	P-value
Maternal Race – Asian	0.14	0.73	1.15 (0.23 - 4.39)	0.85
Maternal Race – Black or African American	0.14	0.33	1.15 (0.59 – 2.18)	0.68
Maternal Race – Declined	0.40	0.34	1.50 (0.76 - 2.91)	0.23
Maternal Race – White	-0.02	0.29	0.98 (0.54 - 1.74)	0.95
Maternal Ethnicity – Declined	-0.59	0.47	0.56 (0.22 - 1.36)	0.21
Maternal Ethnicity – Not Hispanic or Latino or Spanish Origin	-0.28	0.31	0.75 (0.41 – 1.37)	0.36

[a] Prevalence of elevated M-CHAT score in COMBO-EHR RSCH among female children unexposed to SARS-CoV-2 is 0.19

[b] 95% CI could not be estimated due to small sample size in this predictor group.

[c] Prevalence of elevated M-CHAT score in COMBO-EHR among male children unexposed to SARS-CoV-2 is 0.38

© 2024 Firestein MR et al. *JAMA Network Open.*

eTable 14. SARS-CoV-2 Exposure and M-CHAT-R Positive Screenings in Ethnicity Subgroups (Hispanic/Latino or Not Hispanic/Latino; *COMBO-EHR)*

Predictor	B	SE	Odds Ratio 95% CI	P-value
Hispanic/Latino Ethnicity (n=774[a])				
SARS-CoV-2 Infection in Pregnancy	-0.83	0.30	0.44 (0.23 - 0.77)	0.006
Age at M-CHAT administration (months)	-0.05	0.03	0.95 (0.90 - 1.00)	0.05
Gestational age (weeks)	-0.11	0.04	0.89 (0.82 - 0.98)	0.01
Maternal age at delivery	-0.03	0.01	0.97 (0.94 - 1.00)	0.06
Infant sex assigned at birth (Male)	0.51	0.17	1.67 (1.19 - 2.35)	0.003
Insurance (Medicaid)	0.36	0.34	1.43 (0.75 - 2.93)	0.30
Maternal Race – American Indian or Alaska Native[c]	14.13	535.41	NA	0.98
Maternal Race – Black or African American	-0.10	0.33	0.90 (0.46 - 1.69)	0.76
Maternal Race – Declined	0.59	0.28	1.80 (1.04 - 3.08)	0.03
Maternal Race – White	0.18	0.23	1.19 (0.75 - 1.87)	0.44
Not Hispanic/Latino Ethnicity (n=270[b])				
SARS-CoV-2 Infection in Pregnancy	-1.35	0.93	0.26 (0.03 - 1.24)	0.15
Age at M-CHAT administration (months)	0.01	0.05	1.01 (0.92 - 1.12)	0.80
Gestational age (weeks)	-0.21	0.07	0.81 (0.70 - 0.92)	0.001
Maternal age at delivery	0.05	0.03	1.05 (0.99 - 1.12)	0.09
Infant sex assigned at birth (Male)	0.67	0.37	1.96 (0.96 - 4.13)	0.07
Insurance (Medicaid)	0.74	0.48	2.10 (0.83 - 5.52)	0.12
Maternal Race – American Indian or Alaska Native[c]	-16.70	2149.33	NA	0.99
Maternal Race – Asian	-0.58	0.77	0.56 (0.11 - 2.50)	0.45

© 2024 Firestein MR et al. *JAMA Network Open.*

Predictor	B	SE	Odds Ratio 95% CI	P-value
Maternal Race – Black or African American	-0.05	0.53	0.95 (0.35 - 2.80)	0.92
Maternal Race – Declined[c]	-16.27	1192.86	NA	0.99
Maternal Race – White	-1.41	0.70	0.24 (0.06 - 0.96)	0.04

[a] Prevalence of elevated M-CHAT scores in COMBO-EHR Hispanic/Latino group unexposed to SARS-CoV-2 is 0.27
[b] Prevalence of elevated M-CHAT scores in COMBO-EHR Not Hispanic/Latino group unexposed to SARS-CoV-2 is 0.18
[c] 95% CI could not be estimated due to small sample size in this predictor group.

© 2024 Firestein MR et al. *JAMA Network Open.*

**eTable 15. SARS-CoV-2 Exposure and M-CHAT-R Positive Screenings in Commercial and Medicaid Insured Subgroups (COMBO-EHR)**				
**Predictor**	**B**	**SE**	**Odds Ratio 95% CI**	**P-value**
Medicaid Insurance (n=890[a])				
SARS-CoV-2 Infection in Pregnancy	-0.95	0.30	0.39 (0.21-0.68)	0.002
Age at M-CHAT administration (months)	-0.03	0.02	0.97 (0.92 – 1.02)	0.18
Maternal age at delivery	-0.01	0.01	0.99 (0.96 – 1.02)	0.46
Infant Sex assigned at birth (Male)	0.51	0.16	1.67 (1.22 – 2.29)	0.001
Gestational Age	-0.12	0.04	0.88 (0.82 – 0.95)	0.002
Maternal Race – American Indian or Alaska Native[b]	0.51	1.31	1.67 (0.07 – 20.85)	0.70
Maternal Race – Asian	0.02	1.14	1.02 (0.05 – 7.09)	0.99
Maternal Race – Black or African American	0.30	0.27	1.35 (0.79 – 2.28)	0.27
Maternal Race – Declined	0.56	0.27	1.75 (1.02 – 2.97)	0.04
Maternal Race – White	0.16	0.24	1.18 (0.73 – 1.86)	0.49
Maternal Ethnicity - Declined	-0.65	0.43	0.52 (0.22 – 1.16)	0.12
Maternal Ethnicity – Not Hispanic or Latino	-0.30	0.28	0.74 (0.42 – 1.28)	0.29
Commercial Insurance(n=237[c])				
SARS-CoV-2 Infection in Pregnancy	-0.62	0.82	0.54 (0.08 – 2.26)	0.45
Age at M-CHAT administration (months)	-0.09	0.07	0.91 (0.80 -1.04)	0.19
Maternal age at delivery	-0.02	0.04	0.98 (0.91 – 1.05)	0.53
Infant Sex assigned at birth (Male)	0.75	0.43	2.13 (0.94 – 5.11)	0.08

© 2024 Firestein MR et al. *JAMA Network Open.*

Predictor	B	SE	Odds Ratio 95% CI	P-value
Gestational Age	-0.16	0.08	0.85 (0.72 – 1.01)	0.05
Maternal Race – American Indian or Alaska Native[b]	-12.70	882.74	NA	0.99
Maternal Race – Asian	-1.18	0.82	0.31 (0.05 – 1.44)	0.15
Maternal Race – Black or African American	-0.90	0.73	0.41 (0.09 – 1.62)	0.22
Maternal Race – Declined	-0.67	0.80	0.51 (0.10 – 2.29)	0.40
Maternal Race – White	-1.39	0.61	0.25 (0.07 – 0.80)	0.02
Maternal Ethnicity - Declined	-0.07	0.81	0.94 (0.19 – 4.74)	0.94
Maternal Ethnicity – Not Hispanic or Latino	-0.19	0.54	0.83 (0.29 – 2.47)	0.73

[a] Prevalence of elevated M-CHAT score in COMBO-EHR SARS-CoV-2 unexposed group among those insured by Medicaid is 0.27

[b] 95% CI lower was not able to be estimated due to small sample size.

[c] Prevalence of elevated M-CHAT score in COMBO-EHR SARS-CoV-2 unexposed group among those with Commercial Insurance is 0.14

© 2024 Firestein MR et al. *JAMA Network Open.*

eTable 16. SARS-CoV-2 Exposure and M-CHAT-R Positive Screenings in Preterm and Full-Term Subgroup (*COMBO-RSCH)*				
**Predictor**	**B**	**SE**	**Odds Ratio (95% CI)**	**P-value**
Preterm Subgroup (n=27[a])				
SARS-CoV-2 Infection in pregnancy	0.46	2.61	1.59 (0.02, 3069.97)	0.86
Age at M-CHAT administration (months)	-1.02	1.65	0.36 (<0.01, 7.13)	0.54
Maternal age at delivery	0.76	0.71	2.13 (0.84, 25.06)	0.29
Infant sex assigned at birth (Male)	-0.75	2.13	0.47 (<0.01, 24.78)	0.72
Insurance (Medicaid)	26.71	5136.96	NA(NA)	1.00
Maternal Race – American Indian or Alaska Native[b]	NA	NA	NA	NA
Maternal Race – Asian[b]	NA	NA	NA	NA
Maternal Race – Black or African American[b]	NA	NA	NA	NA
Maternal Race – Declined[b]	NA	NA	NA	NA
Maternal Race – Native Hawaiian or Other Pacific Islander[b]	NA	NA	NA	NA
Maternal Race – White[b]	NA	NA	NA	NA
Maternal Ethnicity – Declined[b]	NA	NA	NA	NA
Maternal Ethnicity – Not Hispanic or Latino[b]	NA	NA	NA	NA
Maternal Ethnicity – Unknown[b]	NA	NA	NA	NA
Full-Term Subgroup (n=275[d])				
SARS-CoV-2 Infection in pregnancy	-0.55	0.38	0.58 (0.26, 1.19)	0.15
Age at M-CHAT administration (months)	-0.26	0.23	0.77 (0.44, 1.12)	0.26
Maternal age at delivery	-0.07	0.03	0.93 (0.87, 0.99)	0.03
Infant sex assigned at birth (Male)	0.55	0.36	1.73 (0.87, 3.58)	013
Insurance (Medicaid)	0.23	0.43	1.26 (0.54, 2.99)	0.60
Maternal Race – American Indian or Alaska Native[c]	-14.09	1669.44	NA	0.99
Maternal Race – Asian	0.91	0.84	2.48 (0.42, 12.29)	0.28

© 2024 Firestein MR et al. *JAMA Network Open.*

Predictor	B	SE	Odds Ratio (95% CI)	P-value
Maternal Race – Black or African American	0.62	0.680.68	1.85 (0.46, 6.93)1.	0.37
Maternal Race – Declined	-1.08	0.70	0.34 (0.08, 1.24)	0.12
Maternal Race – Native Hawaiian or Pacific Islander[c]	16.54	2399.54	NA	0.990.
Maternal Race – White	0.30	0.46	1.35 (0.54, 3.33)	0.52
Maternal Ethnicity - Declined	0.49	0.66	1.63 (0.44, 6.04)	0.46
Maternal Ethnicity – Not Hispanic or Latino	-0.66	0.50	0.52 (0.19, 1.37)	0.18
Maternal Ethnicity – Unknown[c]	-12.96-14.55	1455.41699.54	NA	0.990.99

[a] Prevalence of elevated M-CHAT score in COMBO-RSCH Preterm unexposed group is 0.20
[b] Due to small sample size, models could not be estimated with race and ethnicity as covariates so they were omitted.
[c] OR and/or 95% CI could not be estimated due to group size of this predictor.
[d] Prevalence of elevated M-CHAT score in COMBO-RSCH Full term unexposed group is 0.20

© 2024 Firestein MR et al. *JAMA Network Open.*

eTable 17. SARS-CoV-2 Exposure and M-CHAT-R Positive Screenings in Female and Male Subgroups (*COMBO-RSCH)*				
**Predictor**	**B**	**SE**	**Odds Ratio (95% CI)**	**P-value**
Females (n=127[a])				
SARS-CoV-2 Infection in pregnancy	-0.88	0.78	0.42 (0.08, 1.76)	0.26
Age at M-CHAT administration (months)	-0.68	0.48	0.51 (0.15, 1.05)	0.16
Maternal age at delivery	-0.27	0.09	0.76 (0.62, 0.89)	<0.01
Insurance (Medicaid)	0.28	0.84	1.32 (0.25, 7.26)	0.74
Gestational Age	-0.26	0.22	0.77 (0.49, 1.20)	0.24
Maternal Race – American Indian or Alaska Native[b]	-14.34	2786.96	NA	0.99
Maternal Race – Asian[b]	-15.61	2161.95	NA	0.99
Maternal Race – Black or African American	0.66	1.07	1.94 (0.20, 15.82)	0.54
Maternal Race – Declined	-1.81	1.64	0.16 (<0.01, 2.47)	0.27
Maternal Race – White	0.31	0.94	1.37 (0.21, 9.06)	0.74
Maternal Ethnicity - Declined	0.73	1.75	2.08 (0.05, 88.94)	0.68
Maternal Ethnicity – Not Hispanic or Latino	-1.11	1.01	0.33 (0.04, 2.32)	0.27
Maternal Ethnicity – Unknown[b]	-14.72	3956.18	NA	1.00
Males (n=175[c])				
SARS-CoV-2 Infection in pregnancy	-0.73	0.46	0.48 (0.19, 1.15)	0.11
Age at M-CHAT administration (months)	-0.28	0.31	0.75 (0.37, 1.21)	0.35
Maternal age at delivery	-0.02	0.04	0.98 (0.90, 1.06)	0.56
Insurance (Medicaid)	0.55	0.52	1.73 (0.64, 4.91)	0.29
Gestational Age	-0.22	0.16	0.80 (0.58, 1.08)	0.15

© 2024 Firestein MR et al. *JAMA Network Open.*

Predictor	B	SE	Odds Ratio (95% CI)	P-value
Maternal Race – American Indian or Alaska Native [b]	16.04	1455.40	NA	0.99
Maternal Race – Asian	1.77	0.97	5.87 (0.81, 39.86)	0.07
Maternal Race – Black or African American	0.37	0.86	1.45 (0.23, 7.45)	0.67
Maternal Race – Declined	-0.82	0.83	0.44 (0.08, 2.07)	0.32
Maternal Race – Native Hawaiian or Pacific Islander [b]	16.22	1455.40	NA	0.99
Maternal Race – White	0.68	0.55	1.98 (0.68, 6.00)	0.22
Maternal Ethnicity - Declined	0.66	0.76	1.93 (0.43, 8.78)	0.39
Maternal Ethnicity – Not Hispanic or Latino	-0.67	0.57	0.51 (0.16, 1.57)	0.24

[a] Prevalence of elevated M-CHAT score in COMBO-RSCH female infant unexposed group is 0.12
[b] OR and/or 95% CI not able to be estimated due to small group size in this predictor.
[c] Prevalence of elevated M-CHAT score in COMBO-RSCH male infant unexposed group is 0.25

© 2024 Firestein MR et al. *JAMA Network Open.*

eTable 18. SARS-CoV-2 Exposure and M-CHAT-R Positive Screenings among mothers with Hispanic/Latino and Non-Hispanic/Latino Ethnicity (*COMBO-RSCH)*				
**Predictor**	**B**	**SE**	**Odds Ratio 95% CI**	**P-value**
Hispanic/Latino Ethnicity (n=132[a])				
SARS-CoV-2 Infection in Pregnancy	-0.67	0.52	0.51 (0.17, 1.39)	0.20
Age at M-CHAT administration (months)	-0.86	0.42	0.42 (0.17, 0.86)	0.04
Gestational age (weeks)	-0.62	0.23	0.54 (0.34, 0.82)	0.01
Maternal age at delivery	-0.11	0.05	0.90 (0.81, 0.98)	0.03
Infant sex assigned at birth (Male)	0.15	0.50	1.16 (0.44, 3.15)	0.77
Insurance (Medicaid)	1.04	0.62	2.82 (0.90, 10.55)	0.09
Maternal Race – American Indian or Alaska Native	1.15	1.84	3.17 (0.07, 145.31)	0.53
Maternal Race – Black or African American	-1.21	1.27	0.30 (0.01, 2.72)	0.34
Maternal Race – Declined[c]	-17.20	1647.48	NA	0.99
Maternal Race – Native Hawaiian or Other Pacific Islander[c]	18.25	6522.64	NA	1.00
Maternal Race – White	1.24	0.56	3.47 (1.19, 10.72)	0.03
Not Hispanic/Latino Ethnicity (n=127[b])				
SARS-CoV-2 Infection in Pregnancy	-1.29	0.83	0.28 (0.04, 1.17)	0.12
Age at M-CHAT administration (months)	-0.42	0.66	0.66 (0.15, 0.90)	0.52
Gestational age (weeks)	-0.09-	0.23	0.92 (0.58, 1.44)	0.70
Maternal age at delivery	-0.06	0.07	0.94 (0.81, 1.09)	0.41
Infant sex assigned at birth (Male)	1.39	0.72	4.02 (1.11, 19.93)	0.05
Insurance (Medicaid)	-0.32	0.31	0.27 (0.02, 2.88)	0.31
Maternal Race – American Indian or Alaska Native[c]	-15.08	3956.18	NA	1.00
Maternal Race – Asian	-0.76	1.24	0.47 (0.04, 5.72)	0.54

© 2024 Firestein MR et al. *JAMA Network Open.*

Predictor	B	SE	Odds Ratio 95% CI	P-value
Maternal Race – Black or African American	0.04	1.14	1.04 (0.12, 11.70)	0.97
Maternal Race – Declined[c]	-16.12	1799.20	NA	0.99
Maternal Race – White	-1.47	1.00	0.23 (0.03, 2.00)	0.14

[a] Prevalence of elevated M-CHAT scores in COMBO-EHR Hispanic/Latino group unexposed to SARS-CoV-2 is 0.25
[b] Prevalence of elevated M-CHAT scores in COMBO-EHR Hispanic/Latino group unexposed to SARS-CoV-2 is 0.15
[c] Estimate of 95% CI could not be estimated due to small sample size in this predictor group.

© 2024 Firestein MR et al. *JAMA Network Open.*

eTable 19. SARS-CoV-2 Exposure and M-CHAT-R Positive Screenings in Commercial and Medicaid Insured Subgroups (COMBO-RSCH)

Predictor	B	SE	Odds Ratio 95% CI	P-value
Medicaid Insurance (n=126[a])				
SARS-CoV-2 Infection in Pregnancy	-1.27	0.58	0.28 (0.08, 0.82)	0.03
Age at M-CHAT administration (months)	-0.42	0.32	0.66 (0.32, 1.10)	0.20
Maternal age at delivery	-0.13	0.05	0.88 (0.79, 0.96)	0.01
Infant Sex assigned at birth (Male)	0.05	0.51	1.05 (0.39, 2.91)	0.92
Gestational Age	-0.45	0.19	0.63 (0.43, 0.92)	0.02
Maternal Race – American Indian or Alaska Native[b]	16.79	2399.54	NA	0.99
Maternal Race – Black or African American	-0.40	1.04	0.67 (0.07, 4.75)	0.70
Maternal Race – Declined	-0.87	1.00	0.42 (0.04, 2.41)	0.38
Maternal Race – Native Hawaiian or Other Pacific Islander[b]	16.17	2399.54	NA	0.99
Maternal Race – White	1.32	0.61	3.76 (1.15, 12.98)	0.03
Maternal Ethnicity - Declined	0.82	1.12	2.27 (0.25, 24.04)	0.46
Maternal Ethnicity – Not Hispanic or Latino	-1.59	0.98	0.20 (0.03, 1.19)	0.11
Maternal Ethnicity – Unknown[b]	-15.14	2399.54	NA	0.99
Commercial Insurance(n=x176[c])				
SARS-CoV-2 Infection in Pregnancy	-0.38	0.54	0.68 (0.22, 1.89)	0.48
Age at M-CHAT administration (months)	-0.29	0.52	0.75 (0.22, 1.66)	0.58
Maternal age at delivery	-0.08	0.06	0.92 (0.81, 1.04)	0.20
Infant Sex assigned at birth (Male)	1.12	0.56	3.07 (1.08, 10.29)	0.05
Gestational Age	-0.05	0.19	0.95 (0.65, 1.40)	0.79
Maternal Race – American Indian or Alaska Native[b]	-13.06	1028.10	NA	0.99

© 2024 Firestein MR et al. *JAMA Network Open.*

Predictor	B	SE	Odds Ratio 95% CI	P-value
Maternal Race – Asian	0.26	0.96	1.29 (0.18, 3.38)	0.79
Maternal Race – Black or African American	0.94	0.96	2.09 (0.29, 13.96)	0.45
Maternal Race – Declined	-1.75	1.14	0.17 (0.01, 1.47)	0.13
Maternal Race – White	-0.62	0.73	0.54 (0.13, 2.35)	0.39
Maternal Ethnicity - Declined	0.94	0.99	2.56 (0.36, 18.80)	0.34
Maternal Ethnicity – Not Hispanic or Latino	0.15	0.69	1.17 (0.32, 4.89)	0.82

[a] Prevalence of elevated M-CHAT score in COMBO-RSCH SARS-CoV-2 unexposed group among those insured by Medicaid is 0.28

[b] Estimate of 95% CI lower limit not able to be estimated due to small sample size.

[c] Prevalence of elevated M-CHAT score in COMBO-RSCH SARS-CoV-2 unexposed group among those with Commercial Insurance is 0.14

© 2024 Firestein MR et al. *JAMA Network Open.*
