# Supplementary material for: Positive Autism Screening Rates in Toddlers Born During the COVID-19 Pandemic
Source: JAMA Netw Open. 2024 Sep 23;7(9):e2435005. doi: 10.1001/jamanetworkopen.2024.35005 (PMC11420691; doi:10.1001/jamanetworkopen.2024.35005)
Supplement: Supplement 2. — Data Sharing Statement [file jamanetwopen-e2435005-s002.pdf]

# Data Sharing Statement

Firestein. Positive Modified Checklist for Autism in Toddlers-Revised Screening Rates in Pandemic-Born Children. *JAMA Netw Open*. Published September 23, 2024. doi:10.1001/jamanetworkopen.2024.35005

**Data**
**Data available:** No

**Additional Information**
**Explanation for why data not available:** Data contains HIPAA protected information.
